# Secreted indicators of androgen receptor activity in breast cancer pre-clinical models

**DOI:** 10.1186/s13058-021-01478-9

**Published:** 2021-11-04

**Authors:** Toru Hanamura, Jessica L. Christenson, Kathleen I. O’Neill, Emmanuel Rosas, Nicole S. Spoelstra, Michelle M. Williams, Jennifer K. Richer

**Affiliations:** Department of Pathology, University of Colorado, Anschutz Medical Campus, 12800 E. 19th Ave., Aurora, CO 80045 USA

**Keywords:** Breast cancer, Androgen signal, Androgen receptor, Serum factor, *KLK3*, *AZGP1*, *PIP*, Prostate specific antigen (PSA), Zinc-alpha-2-glycoprotein (ZAG), Prolactin induced protein (PIP)

## Abstract

**Purpose:**

Accumulating evidence has attracted attention to the androgen receptor (AR) as a biomarker and therapeutic target in breast cancer. We hypothesized that AR activity within the tumor has clinical implications and investigated whether androgen responsive serum factors might serve as a minimally invasive indicator of tumor AR activity.

**Methods:**

Based on a comprehensive gene expression analysis of an AR-positive, triple negative breast cancer patient-derived xenograft (PDX) model, 163 dihydrotestosterone (DHT)-responsive genes were defined as an androgen responsive gene set. Among them, we focused on genes that were DHT-responsive that encode secreted proteins, namely *KLK3*, *AZGP1* and *PIP*, that encode the secreted factors prostate specific antigen (PSA), zinc-alpha-2-glycoprotein (ZAG) and prolactin induced protein (PIP), respectively. Using AR-positive breast cancer cell lines representing all breast cancer subtypes, expression of candidate factors was assessed in response to agonist DHT and antagonist enzalutamide. Gene set enrichment analysis (GSEA) was performed on publically available gene expression datasets from breast cancer patients to analyze the relationship between genes encoding the secreted factors and other androgen responsive gene sets in each breast cancer subtype.

**Results:**

Anti-androgen treatment decreased proliferation in all cell lines tested representing various tumor subtypes. Expression of the secreted factors was regulated by AR activation in the majority of breast cancer cell lines. In GSEA, the candidate genes were positively correlated with an androgen responsive gene set across breast cancer subtypes.

**Conclusion:**

*KLK3*, *AZGP1* and *PIP* are AR regulated and reflect tumor AR activity. Further investigations are needed to examine the potential efficacy of these factors as serum biomarkers.

**Supplementary Information:**

The online version contains supplementary material available at 10.1186/s13058-021-01478-9.

## Introduction

Among women worldwide, breast cancer is the most frequently diagnosed cancer and one of the major causes of cancer-related mortality [1], and although much more rare (< 1% of all breast cancer cases), it can afflict men as well [2]. Systemic therapy plays a major role in the treatment of both early- and late-stage disease. Therapies are selected based on the biological characteristics of the tumor, namely subtypes classified by the expression of estrogen receptor alpha (ER), progesterone receptor (PR), and amplification of the gene encoding the human epidermal growth factor receptor 2 (HER2) [3–6]. While tumor biology-based selection of systemic therapy has significantly improved the prognosis of breast cancer in the past decade, relapse is still not uncommon, regardless of subtype [3, 5]. Since tumor biological characteristics can change as a result of treatment, for optimal subsequent treatment selection, it is critical that less invasive biomarkers be identified that reflect tumor biology that can be evaluated over time during disease treatment and progression [7].

Androgen receptor (AR) is a transcription factor activated by androgens, such as dihydrotestosterone (DHT) in women and testosterone in men [8] and is expressed at various levels across all breast cancer subtypes [9]. While AR is associated with an overall favorable prognosis among women with ER-positive tumors [10, 11], AR, like ER itself, can support breast cancer progression. For example, we previously found that proliferation of MCF-7 depends on AR signal, and the AR antagonist enzalutamide (Enza) inhibits tumor growth of MCF7 xenografts [12]. Also, AR contributes to ER-targeted therapy resistance mechanisms [13, 14], and switching from ER-dependence to AR-dependence is hypothesized to be a mechanism of resistance to ER-directed endocrine therapies such as tamoxifen and aromatase inhibitors [7, 12, 15–18]. AR also plays a role in anchorage-independent cell survival and cancer stem cell-like characteristics including tumor initiation *in-vivo* [18–22]. Based on these findings, both ongoing and completed clinical trials have been initiated to test the efficacy of AR-targeted therapies, such as bicalutamide and Enza, alone and in combination with other agents in both ER-positive and -negative breast cancer [15, 23]. Although there is no established predictive marker for response to AR-targeted therapy in breast cancer, Enza plus the aromatase inhibitor exemestane was shown to be effective in patients with ER-positive advanced breast cancers with a gene signature-based biomarker indicating AR activation [24], and both bicalutamide and enzalutamide showed efficacy in metastatic AR-positive triple-negative breast cancer (TNBC) [25, 26]. In the ER-negative subset, cell lines corresponding to the luminal AR (LAR) subtype, a TNBC subtype characterized by an AR-regulated gene signature, respond to anti-androgen therapy [21]. In a phase II trial testing the efficacy of Enza in advanced AR-positive TNBC, clinical outcomes appeared superior in patients who had an androgen-driven gene signature [27]. AR expression is associated with poor response to neoadjuvant chemotherapy in ER-positive breast cancer [28], and patients with AR-positive or LAR breast cancer also have poor pathological clinical response to neoadjuvant chemotherapy when compared to other TNBC [29, 30]. Thus, being able to predict AR activity within the tumor via a minimally invasive method may enlighten therapeutic selection [8, 31]. In this study, we explored candidate serum factors that reflect tumor AR activity to support development of noninvasive serum biomarkers of AR activity in breast cancer.

## Materials and methods

### Reagents

DHT (Sigma-Aldrich Corporation, St. Louis, MO, USA) was diluted in 100% ethanol. The AR antagonist enzalutamide (Enza) was provided by Medivation, Inc. (San Francisco, CA, USA) and diluted in dimethyl sulfoxide (DMSO).

### Patient derived xenograft (PDX) study

In our previous study [32], we identified genes regulated by AR in an AR-positive, androgen responsive TNBC PDX model. Using these data, in the present study, 163 genes up-regulated by DHT (*p* < 0.05; fold change ≥ 1.5) were defined as the androgen responsive gene set and used in Gene Set Enrichment Analysis (GSEA). Briefly, mice bearing ER-negative, AR-positive HCI-009 PDX tumors [33] were treated with DHT or vehicle control. Then gene expression profiling of HCI-009 tumors was assessed by RNA-sequencing (RNA-seq), and these data are available in the Gene Expression Omnibus (GEO) database as GSE152246 [32].

### Chromatin immunoprecipitation sequencing

We expanded our data from chromatin immunoprecipitation sequencing (ChIP-seq) previously conducted. These data are available in the GEO database as GSE157862 and reported by the Richer lab [34]. Briefly, serum starved MDA-MB-453 cells were treated for 4 h with vehicle control, DHT (10 nM) or DHT (10 nM) plus Enza (10 μM) followed by chromatin immunoprecipitation using AR antibody. Libraries were sequenced by a next gen- sequencer, read data were processed as described in the previous paper [34]. In the present study, read depth was visualized using Integrative Genomics Viewer software with Human hg19 set as a reference genome.

### qRT-PCR assay

Total RNA from MDA-MB-453 cells cultured for 4-days with vehicle control, DHT (1 nM) alone, DHT (1 nM) plus Enza (20 μM) or Enza (20 μM) alone was extracted using RNeasy Plus Mini Kit (QIAGEN, Germantown, MD, USA). cDNA was synthesized from 1 μg total RNA using qScript cDNA SuperMix (Quanta BioSciences, Inc., Gaithersburg, MD, USA). SYBR Green quantitative gene expression analyses were performed using 7900HT Fast Real-Time PCR System (Applied Biosystems, Waltham, MA, USA) according to the manufacturer’s standard protocol. Primer sequences used in this study are listed in Additional file 8: Table 1. Expression of target genes was quantified using the comparative cycle threshold method normalized to *18S *rRNA. All PCRs were performed in biological and technical triplicates.

### Cell culture and cellular assays

Information about culture medium and authentication of each cell line is summarized in Additional file 9: Table 2. For specified cellular assays, steroid-depleted media was prepared for each cell line using phenol red–free basal medium with dextran-coated charcoal (DCC) stripped serum. Cell line subtyping (ie. ER and HER2 status) was defined based on the reports from Holliday, D. L et al., and Ai, J et al. [21, 35]. For all cellular assays using DHT, cells were cultured in media with the DCC stripped sera for 72 h prior to the assay. For crystal violet assays, cells were plated in 96-well plates and grown for 4-days in indicated conditions. Cells were then fixed in 10% formalin, rinsed in PBS, and stained with 5% crystal violet. Cell bound crystal violet was then dissolved in 10% acetic acid and absorbance was measured at 540 nm. Data were normalized to the mean absorbance of vehicle-treated cells. IC_50_ values for Enza were calculated with GraphPad Prism ver. 8.2.0. The proliferative response of each cell line to DHT was determined as "promotional" or "suppressive" when a significant change was observed at any DHT concentration compared to the control in the same cell line. When neither a promotional or suppressive effect was significant, results were designated as “no response".

### Immunoblotting

Whole-cell lysate and centrifuged conditioned media, consisting of 50 μg protein, were denatured, separated on SDS-PAGE gels and transferred to polyvinylidene fluoride membranes. After blocking in 5% milk in Tris-buffered saline–Tween, membranes were probed overnight at 4℃. Primary antibodies include AR (PG-21, 1:500 dilution; EMD Millipore), PSA (A0562, 1:1000 dilution; DAKO), FKBP5 (#8245, 1:1000 dilution; Cell Signaling), ZAG (sc-13585, 1:1000 dilution; Santa Cruz), GCDFP 15 corresponding to PIP (ab62363, 1:1000 dilution; abcam) and GAPDH (G8795, 1:10,000 dilution; Sigma-Aldrich). For secondary antibodies, IRDye 800 goat anti-rabbit and IRDye 680 goat anti-mouse (LI-COR, Lincoln, NE, USA) were used at 1:10,000. Bands were visualized and quantitated with the Odyssey® CLx Imaging System (LI-COR, Lincoln, NE, USA). Ponceau S staining of the membranes was used as loading control for western blots of conditioned media.

### Analysis of gene expression profile data set

Two publically available gene expression profile data sets, obtained by RNA-seq from breast cancer patient samples, were utilized for analysis in this study: The Cancer Genome Atlas (TCGA) breast cohort [36, 37] (*n* = 1100) and the Sweden Cancerome Analysis Network-Breast (SCAN-B) cohort [38] (*n* = 3273). Clinical and Gene expression data were downloaded from cBioportal (https://www.cbioportal.org/) and Gene Expression Omnibus (https://www.ncbi.nlm.nih.gov/geo/), respectively. All mRNA expression values were log2 transformed prior to analysis. Definitions of clinicopathological characteristics are described in their original papers [33, 34]. Subtypes are classified as follows based on receptor status assessed by immunohistochemistry; ER+/HER2− as Luminal, ER+/HER2+ as Luminal HER2, ER−/HER2+ as HER2, ER−/HER2− as TNBC. Cases showing ER−/PgR+ (progesterone receptor-positive) were defined as unknown subtype. Gene set enrichment analysis (GSEA) [39, 40] was performed to compare specified gene sets using GSEA software v3.0 with the number of permutations set at 1000 and permutation type set as phenotype. Expression values of indicated genes or subtype were used as phenotype labels and Pearson's correlation or Signal-2-Noise was set as Metrics for Ranking Genes. Thresholds for nominal *p* value and FDR *q* value were set at < 0.05 and < 0.25, respectively. Using the TNBC type online subtyping tool (http://cbc.mc.vanderbilt.edu/tnbc/) [41], IHC defined TNBC samples (SCAN-B; *n* = 143, TCGA; *n* = 123) were pre-processed by excluding possible ER-positive samples at the transcriptome level (SCAN-B; *n* = 15, TNBC; *n* = 8), then classified according to Lehmann's molecular subtypes [21]. Subtypes other than LAR were defined as non-LAR, followed by various analysis in LAR vs non-LAR.

### Statistical analysis

All statistical analyses were performed using the GraphPad Prism ver. 8.2.0 software. For comparison of multiple groups in in-vitro studies including proliferative response to different doses of DHT, statistical significance was tested by one-way ANOVA with Dunnett's multiple comparisons test. For comparison of dose–response curves for cell viability between cell lines, Mixed-effect analysis with Tukey's multiple comparisons test was used. Correlation between protein expression levels and Enza IC_50_ were analyzed by Spearman’s rank correlation coefficient. Correlation between genes was analyzed by Pearson's correlation coefficient. For comparison of gene expression of two groups, unpaired *t*- test was used. The TNBC subtype classification performance by candidate gene expression level was evaluated by receiver operating characteristic (ROC) analysis.

## Results

### DHT-responsive genes encoding secreted proteins in an AR-positive TNBC PDX breast cancer model

**Fig. 1:**
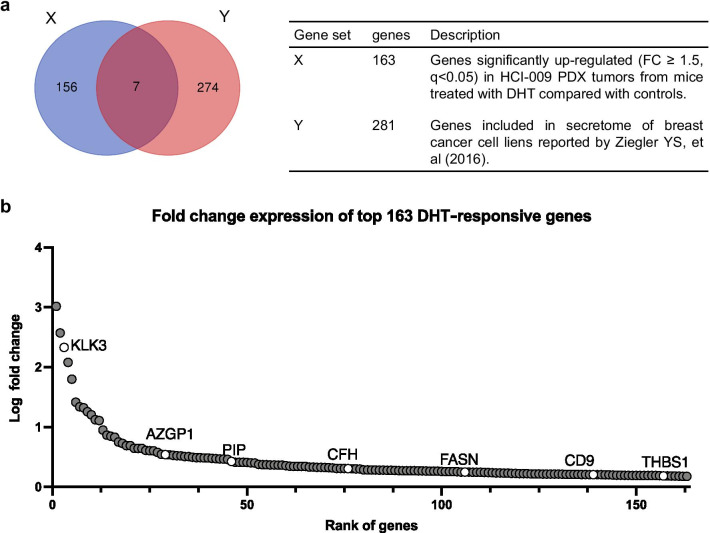
*KLK3*, *AZGP1* and *PIP* are the most DHT-responsive genes that also encode secreted proteins. **a** Gene expression profile of HCI-009 PDX tumor in mice supplemented with cellulose, as a control, or DHT, assessed by RNA-seq. Gene set X consist of163 genes significantly up-regulated (fold change ≥ 1.5, *q* < 0.05) in HCI-009 PDX tumors from mice treated with DHT compared with controls and is defined as the androgen responsive gene set. Gene set Y consist of genes encoding proteins listed in secretome of breast cancer cell lines reported by Ziegler YS, et al. (2016). **b** Fold change expression of genes in DHT treated mice relative to control mice in descending order. Each dot represents a gene, and white circles represent genes encoding secreted proteins and are accompanied by official gene symbols

As described above, in a previous report [32], we explored AR activity in TNBC PDX model HCI-009 that grows in response to DHT in mice and 163 genes up-regulated by DHT were identified. Among these genes, seven encode secreted proteins (Additional file 10: Table 3, [42]) (Fig. 1a). We focused our study on three genes that were most highly upregulated following DHT treatment, specifically, *KLK3*, *AZGP1* and *PIP* that encode the secreted proteins prostate specific antigen (PSA), zinc-Alpha-2-glycoprotein (ZAG) and prolactin induced protein (PIP) (Fig. 1b).

### Gene expression of KLK3, AZGP1 and PIP is directly regulated by AR in MDA-MB-453 cells

**Fig. 2:**
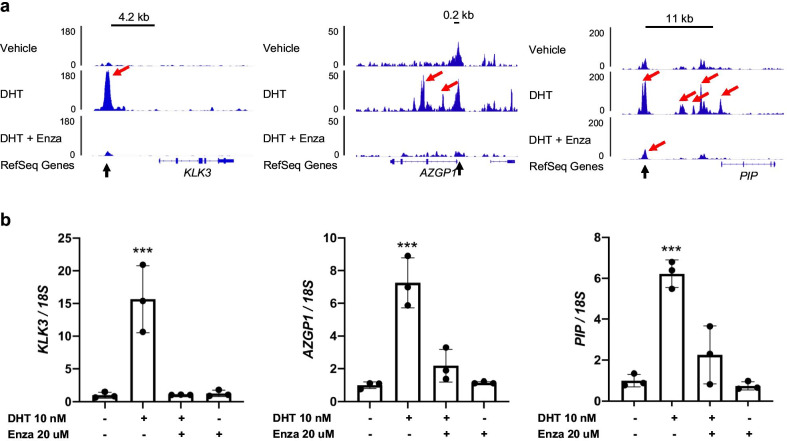
Gene expression of *KLK3*, *AZGP1* and *PIP is* regulated by AR in MDA-MB-453 cells. **a** AR binding on gene loci was assessed by AR-ChIP-seq in MDA-MB-453 breast cancer cells. Gene locus for each candidate gene and read depth for each binding are shown. Red arrows indicate significant AR-binding peaks detected, and black arrows indicate known enhancer regions containing the AR binding motif. **b** Expression of each candidate gene in MDA-MB-453 cells was examined by qRT-PCR after 4-days cultured in indicated conditions. Expression levels were normalized to *18S *rRNA; mean ± standard deviation, ***p < 0.001

MDA-MB-453 breast cancer cells highly express AR [43]. AR-ChIP-seq analysis in MDA-MB-453 cells showed multiple AR binding peaks induced by DHT and/or suppressed by Enza in the gene loci of *KLK3*, *AZGP1* and *PIP*. These included known enhancers containing the AR binding motif (*KLK3* at ~ − 4.2 kb [44], *AZGP1* at ~  + 0.2 kb [45], *PIP* at ~  + 11 kb [46]) (Fig. 2a). qRT-PCR showed that expression of these genes was induced by DHT and suppressed by Enza (Fig. 2b), suggesting that *KLK3*, *AZGP1* and *PIP* may be direct transcriptional targets of AR.

### Protein expression of PSA, ZAG and PIP are regulated by AR in a wide variety of breast cancer cell lines

**Fig. 3:**
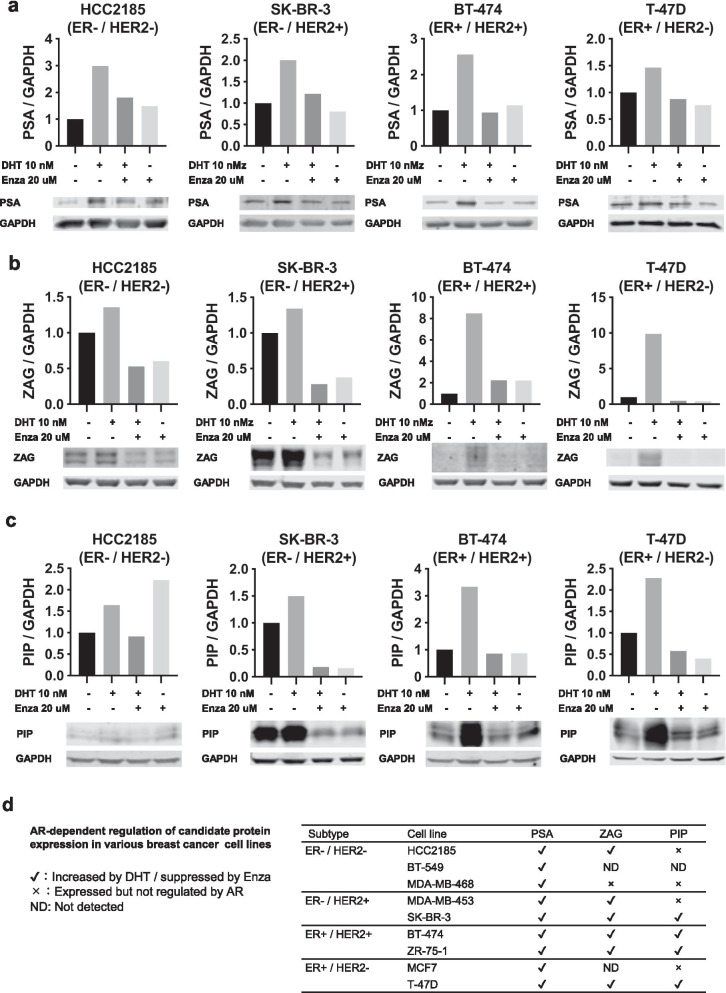
Expression of PSA, ZAG and PIP is regulated by AR in breast cancer cell lines. Protein extracts of whole cell lysates were examined by western blot after 4-days cultured with vehicle control, DHT (10 nM), DHT (10 nM) plus Enza (20 μM) or Enza (20 μM). Each band was quantified by densitometry and the expression levels of PSA (**a**), ZAG (**b**) and PIP (**c**) relative to GAPDH normalized to vehicle control are shown for representative cell lines. Data for the remaining cell lines are shown in Additional file 3: Fig. S3a–c. **d** AR-dependent regulation of candidate protein expression in all tested cell lines are summarized

**Fig. 4:**
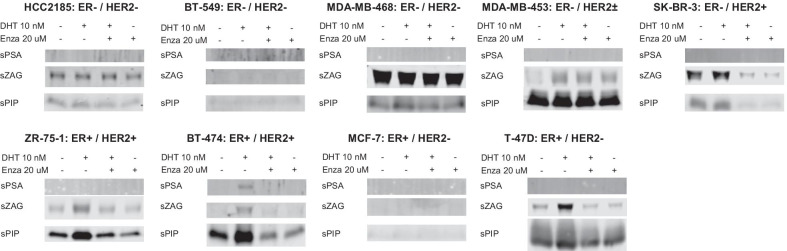
AR-regulated secreted proteins in conditioned media from nine breast cancer cell lines. Conditioned medium (s, secreted proteins) from each cell line was examined by western blot after 4-days culture with vehicle control, DHT (10 nM), DHT (10 nM) plus Enza (20 μM) or Enza (20 μM). Ponceau S staining of the membranes as loading controls for western blots of conditioned medium are shown in Additional file 4: Fig. S4

In order to investigate whether protein expression of the candidate genes was also AR regulated, nine AR expressing breast cancer cell lines were analyzed for PSA, ZAG and PIP protein in whole cell lysates and conditioned media. AR and FKBP5, a known AR-regulated protein, were assessed as well (Additional file 1: Fig. S1). While all cells responded to Enza treatment in a dose-dependent manner, proliferative response to DHT treatment was highly variable (Additional file 2: Fig. S2a–d, Additional file 11: Table 4, Additional file 12: Table 5, Additional file 13: Table 6). Although DHT did not affect cell proliferation in four cell lines, it showed a suppressive effect in three and a promotive effect in two. We did not find any significant correlation between Enza IC_50_ levels and levels of AR or AR-regulated proteins, perhaps due to small sample size (Additional file 2: Fig. S2e). PSA in cell lysates was induced by DHT and suppressed by Enza in all cell lines examined (Fig. 3a, Additional file 3: Fig. S3a). Although ZAG and PIP levels were more variable, ZAG was induced by DHT and suppressed by Enza in 6 out of 9 cell lines (Fig. 3b, Additional file 3: Fig. S3b), while PIP was induced by DHT and suppressed by Enza in 4 out of 9 cell lines (Fig. 3c, Additional file 3: Fig. S3c). On the other hand, analysis of conditioned media showed that while PSA was secreted only in DHT-treated BT-474 cells, ZAG was secreted in all ZAG-expressing cell lines and was induced by DHT and suppressed by Enza in the majority of cell lines and secretion of PIP was obvious in 7 out of 9 cell lines and was induced by DHT and suppressed by Enza in the majority of cell lines (Fig. 4, Additional file 4: Fig. S4).

### Candidate genes are positively correlated with a gene expression profile representative of tumor AR activity

**Fig. 5:**
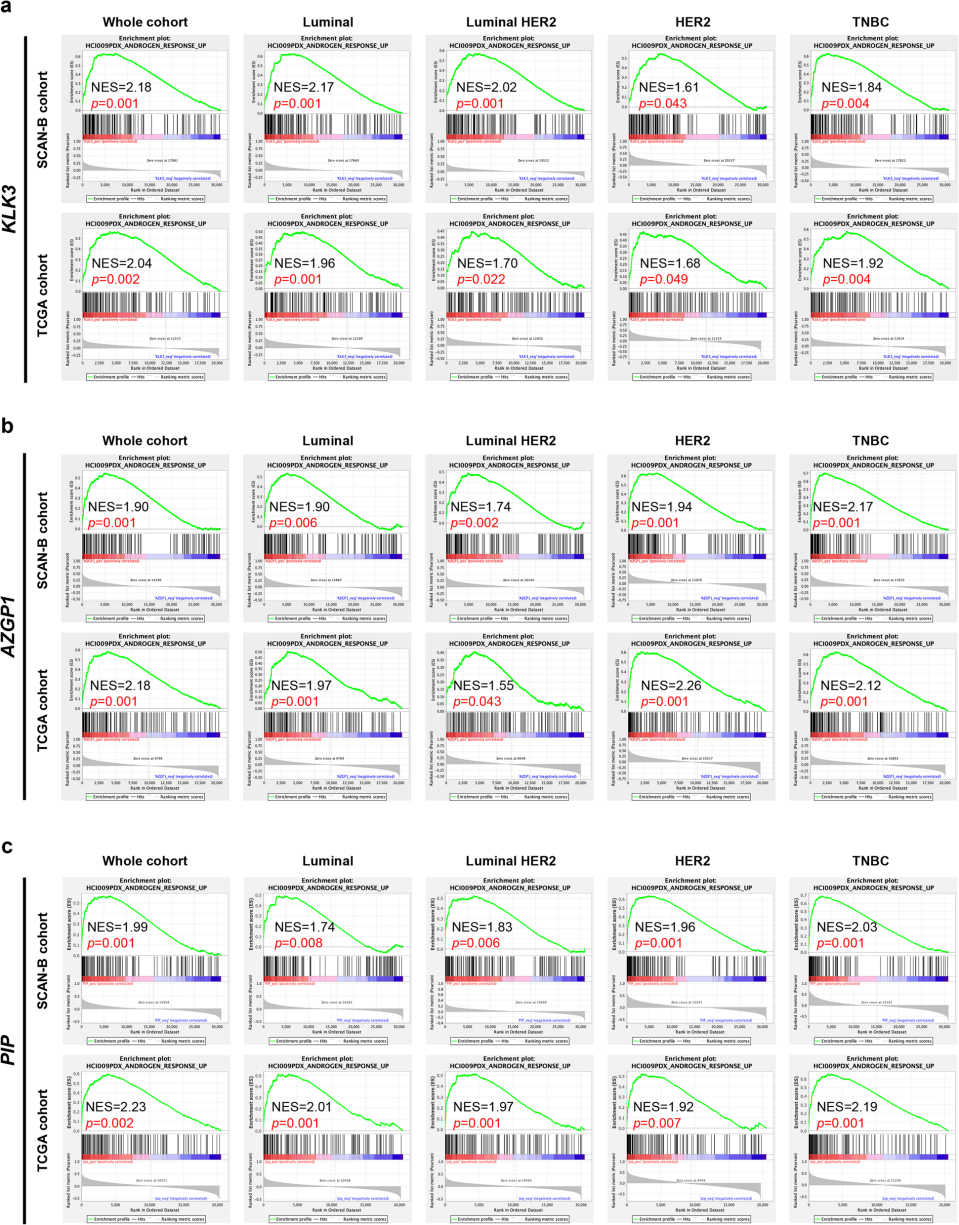
Expression of candidate genes was correlated with a gene expression profile representative of tumor-AR activity. **a**–**c** Gene expression profile data from SCAN-B and TCGA cohorts was applied to GSEA to analyze the relationship between candidate gene expression and the androgen responsive gene set defined in Fig. 1a. Enrichment plots with normal enrichment scores (NES) and *p* values for each gene and breast cancer subtype combination are shown

The correlation between the expression level of candidate genes and that of *AR* was analyzed in the SCANB and TCGA datasets. While *PIP* expression was positively correlated with *AR* expression in all subtypes, *KLK3* and *AZGP1* seemed to be most highly correlated with AR only in the luminal and TNBC subtypes (Additional file 5: Fig. S5a–c). GSEA was then performed on the SCAN-B and TCGA datasets to analyze the relationship between candidate gene expression and the whole androgen responsive gene set defined in HCI009 PDX tumors (Fig. 1a). Expression of *KLK3*, *AZGP1* and *PIP* were shown to be positively correlated with the androgen responsive gene signature in both the SCANB and TCGA cohorts across all subtypes (Fig. 5a–c). As a control comparison, when GSEA is performed using two gene sets representative of estrogen response in breast cancer cells [47], the candidate genes showed no correlation or tended towards being negatively correlated with estrogen responsive genes (Additional file 6: Fig. S6a–f), suggesting that they are indicative of AR activity, but not ER activity.

**Fig. 6:**
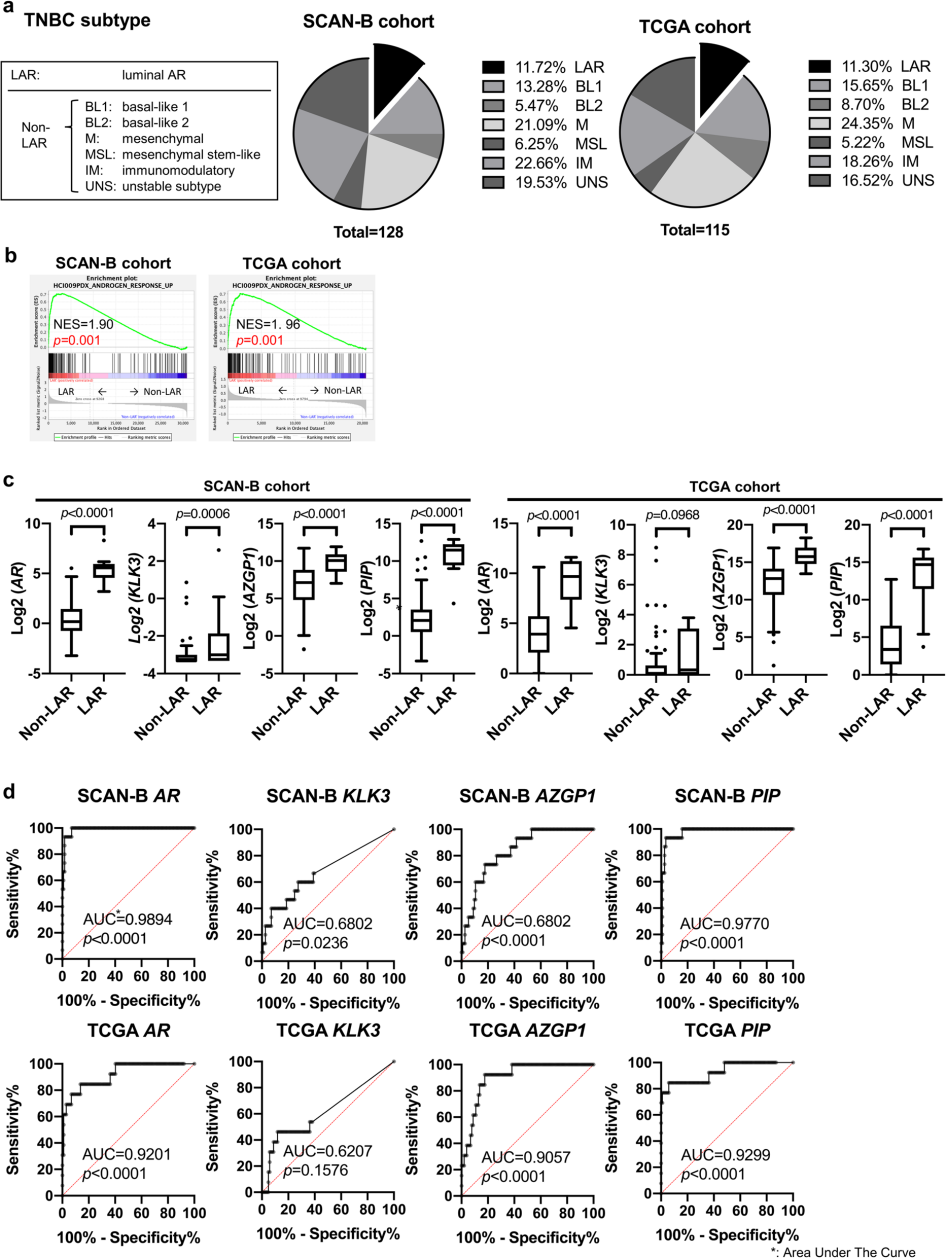
Predictive value of candidate gene expression in TNBC subtypes. **a** TNBC samples from the SCAN-B and TCGA cohorts were classified into Lehmann's molecular subtypes, with samples characterized as being either LAR (luminal AR TNBC subtype) or non-LAR TNBC. The distribution of TNBC subtypes in each cohort is indicated. **b** GSEA was performed on LAR vs non-LAR to examine the relationship between TNBC subtype and the androgen responsive gene set. Enrichment plots with normal enrichment scores (NES) and p values for each cohort. **c** AR and candidate gene expression in LAR vs non-LAR are shown. **d** ROC curves of a given gene as a classifier for LAR with area under the ROC curve (AUC) analyses. The diagonal line denotes the ROC curve of a random classifier of AUC = 0.5. *p* value tests the null hypothesis that the AUC really equals 0.50

According to Lehmann's molecular subtypes of TNBC, IHC defined samples were classified into LAR (*n* = 15 for SCAN-B, *n* = 13 for TCGA) and non-LAR (*n* = 113 for SCAN-B, *n* = 102 for TCGA) (Fig. 6a) [21, 41]. When GSEA was performed on LAR vs non-LAR to examine the relation between TNBC subtype and the androgen responsive gene set, the androgen responsive gene set was shown to be enriched in the LAR samples, as would be expected (Fig. 6b), corresponding to Lehmann's finding that LAR have up-regulated AR activity. We next examined expression of *AR* and candidate genes in the TNBC LAR subtype. *AR*, *AZGP1* and *PIP* were significantly higher in LAR compared to non-LAR TNBC (Fig. 6c). Similarly, A ROC analysis was performed to examine whether these candidate genes can predict the LAR phenotype among TNBC subtypes. Area under the curve (AUC) values showed that *PIP* was as effective as *AR* in distinguishing the LAR TNBC subtype (Fig. 6d).

## Discussion

Recent findings regarding the role of AR in breast cancer [7, 12–17] suggest that estimating AR activity may have clinical utility since trials with both Enzalutamide and Enobosarm [48] showed efficacy in metastatic ER + disease resistant to other endocrine and chemotherapy treatments. Here we identified PSA, ZAG and PIP as candidate biomarkers reflecting tumor AR activity in breast cancer. These factors are all secreted proteins with potential to serve as serum biomarkers [49–53].

We selected three genes encoding secreted proteins most responsive to DHT in an AR-positive TNBC PDX model: *KLK3*, *AZGP1* and *PIP*. In an AR-ChIP-seq analysis of MDA-MB-453 cells, AR binding was demonstrated at androgen response elements upstream of these genes. Other candidate genes among seven androgen responsive genes included *FASN* (fatty acid synthase), *CD9* (CD9) and *THBS1* (thrombospondin 1), which also showed significant AR binding at their gene loci (data not shown).

The majority of AR-regulated gene expression data are from prostate cancer cell lines and PSA is a well-known AR-regulated protein [44, 54, 55]. In breast cancer, previous studies show that DHT can induce PSA expression in cell lines [56–58]. Also, there is evidence in the literature supporting our determination of ZAG [59, 60] and PIP [46, 61, 62] as androgen responsive in breast cancer. In this study, candidate protein expression was analyzed in 9 cell lines representing all breast cancer subtypes, showing that some are more responsive to androgen and some are less so (Figs. 3, 4). GSEA analysis (Fig. 5a–c) suggests that these candidate factors correlate with AR in a considerable number of breast cancer patients across breast cancer subtypes.

In the analysis of anti-proliferative responses of cell lines to Enza, it effectively inhibited growth in all cell lines tested, and IC50 for each cell line averaged 43.8 ± 12.9 μM which is a clinically achievable concentration [20]. For comparison, Enza IC_50_ values for prostate cancer cell lines have been reported to be approximately 10–40 μM [63]. These results suggest that AR-positive breast cancer cells rely on AR function for survival, to some extent, regardless of subtype or proliferative response to DHT (Additional file 2: Fig. S2a–d). These observations are consistent with previous reports showing the pro-tumor roles of AR in multiple breast cancer subtypes [12–14, 16, 18–22, 64, 65]. We theorized that high expression of candidate proteins indicates a higher androgen dependence and responsiveness to Enza. We analyzed correlations between Enza IC_50_ and expression of AR or the candidate proteins in in vitro models (Additional file 2: Fig. S2e), and while statistical significance was not achieved, it is a future direction to test this correlation in clinical trials using AR-targeted therapy and would be particularly useful if PSA, AZGP1 or PIP in serum correlated with AR dependency/response to AR targeting agents.

As shown above, with regard to *KLK3* and *AZGP1*, the response to DHT and Enza in MDA-MB-453 cells was consistent for both mRNA and protein. MDA-MB-453 has such an abundance of PIP protein that the reactivity to DHT and Enza cannot be evaluated properly, but previous reports from other groups using breast cancer cell lines showed that gene and protein expression are linked, with regard to PSA and PIP [46, 58, 66]. We therefore examined the significance of these candidate genes, instead of protein levels, in publically available data sets. *KLK3* and *AZGP1* expression were not strongly correlated with *AR* expression in all breast cancer subtypes (Additional file 5: Fig. S5a, b). However, AR activity cannot be measured solely by *AR* expression, and it is known that activity can be regulated by ligand binding and competing ER [67–71]. However, consistent with our hypothesis, the results of GSEA using the androgen responsive gene set demonstrated that the expression of candidate genes accurately reflects a larger gene signature representing tumor AR activity in all breast cancer subtypes (Fig. 5a–c). Since the candidate genes were identified from the androgen response genes in HCI-009 PDX and GSEA was performed using this androgen response gene set, it is not surprising that candidate genes correlate with this androgen-responsive gene set. However, we also validated this analysis using another androgen-responsive gene set derived from an AR-positive breast cancer cell line MDA-MB-453 [64]. With the exception in *KLK3* expression in some subtypes, candidate genes showed a significant positive correlation with this androgen-responsive gene set (Additional file 7: Fig. S7). When the same analysis was performed on the less strongly expressed -candidate genes, (*FASN*, *CFH*, *CD9* and *THBS1*), only *FASN* showed a correlation with the androgen responsive gene set (data not shown). Interestingly, *AZGP1* and *PIP* were particularly high in the LAR TNBC subtype (Fig. 6c); thus, serum levels may be useful as markers for predicting LAR TNBC AR dependency with high accuracy.

Although it is difficult to accurately quantify the protein levels in conditioned medium by western blot because there are no good internal standards, relative levels suggest that the secreted candidate proteins in the conditioned medium are regulated by AR in many of the cell lines (Fig. 4). Serum PSA is an established biomarker of tumor burden in prostate cancer [72]. Although there is some controversy regarding the differences in PSA expression between normal and breast cancer tissues [73], several groups, including us, have reported that serum PSA levels are higher in breast cancer patients than in healthy women, indicating that tumor derived PSA is detectable in serum of breast cancer patients [52, 55, 74, 75]. Recently, we found that serum PSA levels positively correlated with AR expression in primary tumors [52]. Combined with the present findings, the future clinical use of serum PSA as a biomarker for tumor AR activity in breast cancer is promising. However, in this study, PSA could not be detected in the conditioned medium of most cell lines examined (Fig. 4). In our previous analysis, the positive rate of serum PSA was 36.1% for metastatic breast cancer and 13.3% for early-stage breast cancer. [52], suggesting considerably low levels of serum PSA in most breast cancer cases. Therefore, establishing a more sensitive assay for PSA quantification is necessary for full clinical translation. ZAG expression in breast cancer has been documented and is considered as a potential biomarker for breast carcinoma [50, 76–79] because its expression is detected exclusively in patients with ductal carcinoma when compared to the normal breast tissue of healthy women. Serum ZAG is also significantly higher in breast cancer patients than healthy control patients as well as correlating with disease burden in breast cancer patients [50]. PIP, also known as gross cystic disease fluid protein 15 (GCDFP-15), is commonly used in the clinic as a breast cancer biomarker [80–83] to assist in characterizing metastases of unknown origin. There has also been controversy over the difference in PIP expression between normal and breast cancer tissues. In early studies, PIP was shown to be absent in normal breast epithelium, whereas in breast cancers PIP is frequently expressed [81, 82]. Others have shown that PIP is frequently present in uninvolved breast tissue [84]. However, since highly increased levels of PIP have been detected in the peripheral plasma from patients with primary and metastatic breast cancer in comparison to normal subjects, its significance as a potential serum marker in breast cancer is promising [51, 82, 85]. Taken together, serum levels of these candidate proteins may reflect tumor AR activity. Thus, further studies are needed to determine the relationship between serum levels of these factors and tumor biology. It is possible that these proteins might not have a predictive advantage over the general AR gene signature of tumor tissue itself. However, breast cancer cells change their biological properties as they develop resistance to treatment (reviewed in T. Hanamura et al. [7]). Clinically, in the advanced or metastatic setting, systemic therapy is thought to induce alterations to tumor biology as well [86]. Because gene signature monitoring requires tissue collection that cannot be done easily or often, these candidate secreted proteins are suggested to be useful as less invasive biomarkers that can be assessed at any time point to help monitor drug response or resistance.

Assessments of tumor AR signaling by liquid biopsy have been reported by other groups. Both AR mRNA and protein in circulating tumor cells was investigated and shown to be evaluable in blood samples [87–89]. In recent years, deep-sequencing techniques applied to blood samples have shown that AR pathways are activated in circulating tumor cells from bone-predominant breast cancer [90]. Other groups found that 47% of all *AR* variants in cell-free DNA of breast cancer patients were pathogenic or likely pathogenic [91]. Although, like our three candidate proteins, the predictive value of these *AR* variants needs to be evaluated in future trials.

Finally, AR signals are known to have immunosuppressive effects in *in-vivo* models of various autoimmune diseases, follicular thyroid cancer and colon cancer [92]. ZAG has structural similarities to MHC class I, and analysis of various disease models has shown that it may suppress immune response, but its function in the cancer microenvironment is not clear [79]. PIP plays multiple roles in biology, including fertility, immuno-regulation, anti-microbial activity, and tumor progression [93]. Interestingly, ZAG and PIP can form a complex with each other, suggesting a cooperative role between these two proteins [94], although their biological significance / activity in breast cancer remains to be determined. Thus, we are currently conducting further pre-clinical and clinical evaluations of these proteins and their immune-modulatory potential in breast cancer.

## Conclusion

We identified PSA, ZAG and PIP as candidate biomarkers reflecting tumor AR activity in breast cancer. The expression levels of candidate factors are closely correlated with a gene signature representing tumor AR activity in all breast cancer subtypes. However, the responsiveness to DHT and Enza in cell lines is highly variable. Further verification of the biological significance of these candidate factors will be needed in the future. To establish the potential utility of these secreted proteins as serum-derived indicators of tumor AR activity, further studies are needed to determine the relationship between serum levels of these factors and tumor biology, specifically how they correlate with response to AR targeting agents in clinical trials. These secreted factors may be particularly useful in breast cancer patients with metastatic disease resistant to traditional therapies, where AR targeting drugs are being evaluated in multiple ongoing clinical trials.

## Supplementary Information

**Additional file 1: Fig. S1.** Western blot for AR and FKBP5 in nine breast cancer cell lines. Protein extracts from whole cell lysates were examined by western blot after 4-days cultured with vehicle control, DHT (10 nM), DHT (10 nM) plus Enza (20 μM) or Enza (20 μM).

**Additional file 2: Fig. S2.** AR-expressing breast cancer cells respond to Enza regardless of subtype or proliferative response to DHT. **a** Cell viability was assessed by crystal violet assay after 4-days cultured in full serum media with increasing concentrations of Enza. **b** Shown are Enza IC50 (μM) values summarized by tumor subtype. Table indicates subtype of the cell lines and corresponding Enza IC50 (μM) values. **c** Cell viability was assessed by crystal violet assay after 4-days cultured in hormone-depleted medium supplemented with increasing concentrations of DHT. **d** Shown are Enza IC50 (μM) values summarized by proliferative response to DHT. Table shows the proliferative response to DHT and Enza IC50 (μM) values for each cell line. **e** Expression levels of proteins relative to GAPDH were determined by western blot. Scatter plots examining correlations between protein expression and Enza IC50 levels. Regression lines are indicative of the overall correlation.

**Additional file 3: Fig. S3.** Protein expression of PSA, ZAG and PIP in AR expressing breast cancer cell lines. Protein expression in nine cell lines were examined as shown in Fig. 3. Data for the remaining cell lines not shown in Figure 3 are shown.

**Additional file 4: Fig. S4.** Ponceau S staining of western blot conditioned media membranes. Ponceau S staining of the membranes as loading controls for western blots of conditioned medium (s, secreted proteins).

**Additional file 5: Fig. S5.** Correlation analysis of candidate gene expression and AR using gene expression profile data sets. **a**–**c** Scatter plots show the correlation between expression values for candidate genes and AR, with regression lines, Pearson’s correlation coefficients (*r*) and *p* values.

**Additional file 6: Fig. S6.** Association between candidate gene expression and the gene expression profile representative of tumor estrogen response. **a**–**f** The gene expression profile data from the TCGA and SCAN-B cohorts were applied to GSEA with regard to two gene sets representative of estrogen response in breast cancer cells, DUTERTRE_ESTRADIOL_RESPONSE_6HR_UP (**a**–**c**) and DUTERTRE_ESTRADIOL_ RESPONSE_24HR_UP (**d**–**f**). Enrichment plots with normal enrichment scores (NES) and *p* value for each cohort and subtype are shown.

**Additional file 7: Fig. S7.** Association between candidate gene expression and the gene expression profile representative of tumor androgen response. **a**–**c** The gene expression profile data from the TCGA and SCAN-B cohorts were applied to GSEA with regard to gene sets representative of androgen response in breast cancer cell lines, DOANE_RESPONSE_TO_ANDROGEN_UP. Enrichment plots with normal enrichment scores (NES) and *p* value for each cohort and subtype are shown.

**Additional file 8: Table 1.** Primer sequence used in this study.

**Additional file 9: Table 2.** Growth medium.

**Additional file 10: Table 3.** Genes encoding secreted protein in breast cancer cell lines reported by Ziegler YS, et al.

**Additional file 11: Table 4.** Comparison of proliferative response to Enza between cell lines.

**Additional file 12: Table 5.** Comparison of proliferative response to DHT between cell lines.

**Additional file 13: Table 6.** Comparison of proliferative response to different dose of DHT in same cell lines.

## Data Availability

Request for additional details of the data used in the manuscript can be directed to the corresponding author.

## Abbreviations

AR: Androgen receptor
DHT: Dihydrotestosterone
Enza: Enzalutamide
PDX: Patient derived xenograft
*KLK3*: Kallikrein related peptidase 3
*AZGP1*: Alpha-2-Glycoprotein 1, Zinc-Binding
*PIP*: Prolactin Induced Protein
PSA: Prostate specific antigen
ZAG: Zinc-alpha-2-glycoprotein
GSEA: Gene set enrichment analysis
ER: Estrogen receptor alpha
HER2: Human epidermal growth factor receptor 2
LAR: Luminal AR
TNBC: Triple negative breast cancer
RNA-seq: RNA-sequencing
ChIP-seq: Chromatin immunoprecipitation sequencing
NSE: Normalized enrichment score
GCDFP-15: Gross Cystic Disease Fluid Protein 15

## References

[1] Libson S, Lippman M (2014). A review of clinical aspects of breast cancer. Int Rev Psychiatry (Abingdon, England).

[2] Abdelwahab Yousef AJ (2017). Male Breast cancer: epidemiology and risk factors. Semin Oncol.

[3] Harbeck N, Gnant M (2017). Breast cancer. Lancet (London, England).

[4] Gnant M, Harbeck N, Thomssen C (2017). St. Gallen/Vienna 2017: a brief summary of the consensus discussion about escalation and de-escalation of primary breast cancer treatment. Breast Care (Basel, Switzerland).

[5] Clarke R, Leonessa F, Welch JN, Skaar TC (2001). Cellular and molecular pharmacology of antiestrogen action and resistance. Pharmacol Rev.

[6] Loibl S, Gianni L (2017). HER2-positive breast cancer. Lancet (London, England).

[7] Hanamura T, Hayashi SI (2017). Overcoming aromatase inhibitor resistance in breast cancer: possible mechanisms and clinical applications. Breast Cancer (Tokyo, Japan).

[8] Vasiliou SK, Diamandis EP (2019). Androgen receptor: a promising therapeutic target in breast cancer. Crit Rev Clin Lab Sci.

[9] Collins LC, Cole KS, Marotti JD, Hu R, Schnitt SJ, Tamimi RM (2011). Androgen receptor expression in breast cancer in relation to molecular phenotype: results from the Nurses' Health Study. Mod Pathol.

[10] Hu R, Dawood S, Holmes MD, Collins LC, Schnitt SJ, Cole K (2011). Androgen receptor expression and breast cancer survival in postmenopausal women. Clin Cancer Res.

[11] Park S, Koo JS, Kim MS, Park HS, Lee JS, Lee JS (2011). Androgen receptor expression is significantly associated with better outcomes in estrogen receptor-positive breast cancers. Ann Oncol.

[12] Cochrane DR, Bernales S, Jacobsen BM, Cittelly DM, Howe EN, D'Amato NC (2014). Role of the androgen receptor in breast cancer and preclinical analysis of enzalutamide. Breast Cancer Res BCR.

[13] Fujii R, Hanamura T, Suzuki T, Gohno T, Shibahara Y, Niwa T (2014). Increased androgen receptor activity and cell proliferation in aromatase inhibitor-resistant breast carcinoma. J Steroid Biochem Mol Biol.

[14] De Amicis F, Thirugnansampanthan J, Cui Y, Selever J, Beyer A, Parra I (2010). Androgen receptor overexpression induces tamoxifen resistance in human breast cancer cells. Breast Cancer Res Treat.

[15] Basile D, Cinausero M, Iacono D, Pelizzari G, Bonotto M, Vitale MG (2017). Androgen receptor in estrogen receptor positive breast cancer: Beyond expression. Cancer Treat Rev.

[16] D'Amato NC, Gordon MA, Babbs B, Spoelstra NS, Carson Butterfield KT, Torkko KC (2016). Cooperative dynamics of AR and ER activity in breast cancer. Mol Cancer Res MCR.

[17] Hickey TE, Robinson JL, Carroll JS, Tilley WD (2012). Minireview: the androgen receptor in breast tissues: growth inhibitor, tumor suppressor, oncogene?. Mol Endocrinol (Baltimore, Md).

[18] Williams MM, Spoelstra NS, Arnesen S, O'Neill KI, Christenson JL, Reese J (2021). Steroid hormone receptor and infiltrating immune cell status reveals therapeutic vulnerabilities of ESR1-mutant breast cancer. Can Res.

[19] Barton VN, Christenson JL, Gordon MA, Greene LI, Rogers TJ, Butterfield K (2017). Androgen receptor supports an anchorage-independent, cancer stem cell-like population in triple-negative breast cancer. Can Res.

[20] Barton VN, D'Amato NC, Gordon MA, Lind HT, Spoelstra NS, Babbs BL (2015). Multiple molecular subtypes of triple-negative breast cancer critically rely on androgen receptor and respond to enzalutamide in vivo. Mol Cancer Ther.

[21] Lehmann BD, Bauer JA, Chen X, Sanders ME, Chakravarthy AB, Shyr Y (2011). Identification of human triple-negative breast cancer subtypes and preclinical models for selection of targeted therapies. J Clin Investig.

[22] Ni M, Chen Y, Lim E, Wimberly H, Bailey ST, Imai Y (2011). Targeting androgen receptor in estrogen receptor-negative breast cancer. Cancer Cell.

[23] Barton VN, Gordon MA, Richer JK, Elias A (2016). Anti-androgen therapy in triple-negative breast cancer. Ther Adv Med Oncol.

[24] Krop I, Abramson V, Colleoni M, Traina T, Holmes F, Estevez L (2018). Abstract GS4–07: Results from a randomized placebo-controlled phase 2 trial evaluating exemestane ± enzalutamide in patients with hormone receptor–positive breast cancer. Cancer Res.

[25] Gucalp A, Tolaney S, Isakoff SJ, Ingle JN, Liu MC, Carey LA (2013). Phase II trial of bicalutamide in patients with androgen receptor-positive, estrogen receptor-negative metastatic breast cancer. Clin Cancer Res.

[26] Traina TA, Miller K, Yardley DA, Eakle J, Schwartzberg LS, O'Shaughnessy J (2018). Enzalutamide for the treatment of androgen receptor-expressing triple-negative breast cancer. J Clin Oncol.

[27] Traina TA, Miller K, Yardley DA, O'Shaughnessy J, Cortes J, Awada A (2015). Results from a phase 2 study of enzalutamide (ENZA), an androgen receptor (AR) inhibitor, in advanced AR+ triple-negative breast cancer (TNBC). J Clin Oncol.

[28] Okano M, Oshi M, Butash AL, Asaoka M, Katsuta E, Peng X (2019). Estrogen receptor positive breast cancer with high expression of androgen receptor has less cytolytic activity and worse response to neoadjuvant chemotherapy but better survival. Int J Mol Sci.

[29] Asano Y, Kashiwagi S, Onoda N, Kurata K, Morisaki T, Noda S (2016). Clinical verification of sensitivity to preoperative chemotherapy in cases of androgen receptor-expressing positive breast cancer. Br J Cancer.

[30] Masuda H, Baggerly KA, Wang Y, Zhang Y, Gonzalez-Angulo AM, Meric-Bernstam F (2013). Differential response to neoadjuvant chemotherapy among 7 triple-negative breast cancer molecular subtypes. Clin Cancer Res.

[31] Kono M, Fujii T, Lim B, Karuturi MS, Tripathy D, Ueno NT (2017). Androgen receptor function and androgen receptor-targeted therapies in breast cancer: a review. JAMA Oncol.

[32] Christenson JL, O'Neill KI, Williams MM, Spoelstra NS, Jones KL, Trahan GD (2021). Activity of combined androgen receptor antagonism and cell cycle inhibition in androgen receptor-positive triple-negative breast cancer. Mol Cancer Ther.

[33] DeRose YS, Wang G, Lin YC, Bernard PS, Buys SS, Ebbert MT (2011). Tumor grafts derived from women with breast cancer authentically reflect tumor pathology, growth, metastasis and disease outcomes. Nat Med.

[34] Rosas E, Roberts JT, O'Neill KI, Christenson JL, Williams MM, Hanamura T (2021). A positive feedback loop between TGFβ and androgen receptor supports triple-negative breast cancer anoikis resistance. Endocrinology.

[35] Holliday DL, Speirs V (2011). Choosing the right cell line for breast cancer research. Breast Cancer Res BCR.

[36] Cerami E, Gao J, Dogrusoz U, Gross BE, Sumer SO, Aksoy BA (2012). The cBio cancer genomics portal: an open platform for exploring multidimensional cancer genomics data. Cancer Discov.

[37] Koboldt DC, Fulton R, McLellan M, Schmidt H, Kalicki-Veizer J, McMichael J, Fulton L, Dooling D, Ding L, Mardis E, Wilson R (2012). Comprehensive molecular portraits of human breast tumours. Nature.

[38] Brueffer C, Vallon-Christersson J, Grabau D, Ehinger A, Häkkinen J, Hegardt C (2018). Clinical value of RNA sequencing-based classifiers for prediction of the five conventional breast cancer biomarkers: a report from the population-based Multicenter Sweden Cancerome Analysis Network—breast initiative. JCO Precis Oncol.

[39] Mootha VK, Lindgren CM, Eriksson KF, Subramanian A, Sihag S, Lehar J (2003). PGC-1alpha-responsive genes involved in oxidative phosphorylation are coordinately downregulated in human diabetes. Nat Genet.

[40] Subramanian A, Tamayo P, Mootha VK, Mukherjee S, Ebert BL, Gillette MA (2005). Gene set enrichment analysis: a knowledge-based approach for interpreting genome-wide expression profiles. Proc Natl Acad Sci USA.

[41] Chen X, Li J, Gray WH, Lehmann BD, Bauer JA, Shyr Y (2012). TNBCtype: a subtyping tool for triple-negative breast cancer. Cancer Inform.

[42] Ziegler YS, Moresco JJ, Yates JR, Nardulli AM (2016). Integration of breast cancer secretomes with clinical data elucidates potential serum markers for disease detection, diagnosis, and prognosis. PLoS ONE.

[43] Hall RE, Birrell SN, Tilley WD, Sutherland RL (1994). MDA-MB-453, an androgen-responsive human breast carcinoma cell line with high level androgen receptor expression. Eur J Cancer (Oxford, England: 1990).

[44] Cleutjens KB, van der Korput HA, van Eekelen CC, van Rooij HC, Faber PW, Trapman J (1997). An androgen response element in a far upstream enhancer region is essential for high, androgen-regulated activity of the prostate-specific antigen promoter. Mol Endocrinol (Baltimore, Md).

[45] Cao R, Ke M, Wu Q, Tian Q, Liu L, Dai Z (2019). AZGP1 is androgen responsive and involved in AR-induced prostate cancer cell proliferation and metastasis. J Cell Physiol.

[46] Baniwal SK, Little GH, Chimge NO, Frenkel B (2012). Runx2 controls a feed-forward loop between androgen and prolactin-induced protein (PIP) in stimulating T47D cell proliferation. J Cell Physiol.

[47] Dutertre M, Gratadou L, Dardenne E, Germann S, Samaan S, Lidereau R (2010). Estrogen regulation and physiopathologic significance of alternative promoters in breast cancer. Can Res.

[48] Novel agents show promise against acquired endocrine resistance in ER+ advanced breast cancer. Oncologist. 2021;26(Suppl 3):S15–S6 doi 10.1002/onco.13874. 10.1002/onco.13874 PMC8262303 34173302

[49] Black MH, Giai M, Ponzone R, Sismondi P, Yu H, Diamandis EP (2000). Serum total and free prostate-specific antigen for breast cancer diagnosis in women. Clin Cancer Res.

[50] Bundred NJ, Scott WN, Davies SJ, Miller WR, Mansel RE (1991). Zinc alpha-2 glycoprotein levels in serum and breast fluids: a potential marker of apocrine activity. Eur J Cancer (Oxford, England: 1990).

[51] Haagensen DE, Kister SJ, Panick J, Giannola J, Hansen HJ, Wells SA (1978). Comparative evaluation of carcinoembryonic antigen and gross cystic disease fluid protein as plasma markers for human breast carcinoma. Cancer.

[52] Hanamura T, Ohno K, Hokibara S, Murasawa H, Nakamura T, Watanabe H (2019). Clinical significance of serum PSA in breast cancer patients. BMC Cancer.

[53] Hortobagyi GN, Stemmer SM, Burris HA, Yap YS, Sonke GS, Paluch-Shimon S (2016). Ribociclib as first-line therapy for HR-positive, advanced breast cancer. N Engl J Med.

[54] Cleutjens KB, van Eekelen CC, van der Korput HA, Brinkmann AO, Trapman J (1996). Two androgen response regions cooperate in steroid hormone regulated activity of the prostate-specific antigen promoter. J Biol Chem.

[55] Perez-Ibave DC, Burciaga-Flores CH, Elizondo-Riojas MA (2018). Prostate-specific antigen (PSA) as a possible biomarker in non-prostatic cancer: a review. Cancer Epidemiol.

[56] Magklara A, Grass L, Diamandis EP (2000). Differential steroid hormone regulation of human glandular kallikrein (hK2) and prostate-specific antigen (PSA) in breast cancer cell lines. Breast Cancer Res Treat.

[57] Yu H, Diamandis EP, Zarghami N, Grass L (1994). Induction of prostate specific antigen production by steroids and tamoxifen in breast cancer cell lines. Breast Cancer Res Treat.

[58] Zarghami N, Grass L, Diamandis EP (1997). Steroid hormone regulation of prostate-specific antigen gene expression in breast cancer. Br J Cancer.

[59] Chalbos D, Haagensen D, Parish T, Rochefort H (1987). Identification and androgen regulation of two proteins released by T47D human breast cancer cells. Can Res.

[60] Lopez-Boado YS, Diez-Itza I, Tolivia J, Lopez-Otin C (1994). Glucocorticoids and androgens up-regulate the Zn-alpha 2-glycoprotein messenger RNA in human breast cancer cells. Breast Cancer Res Treat.

[61] Dumont M, Dauvois S, Simard J, Garcia T, Schachter B, Labrie F (1989). Antagonism between estrogens and androgens on GCDFP-15 gene expression in ZR-75-1 cells and correlation between GCDFP-15 and estrogen as well as progesterone receptor expression in human breast cancer. J Steroid Biochem.

[62] Simard J, Hatton AC, Labrie C, Dauvois S, Zhao HF, Haagensen DE (1989). Inhibitory effect of estrogens on GCDFP-15 mRNA levels and secretion in ZR-75-1 human breast cancer cells. Mol Endocrinol (Baltimore, Md).

[63] Smith R, Liu M, Liby T, Bayani N, Bucher E, Chiotti K (2020). Enzalutamide response in a panel of prostate cancer cell lines reveals a role for glucocorticoid receptor in enzalutamide resistant disease. Sci Rep.

[64] Doane AS, Danso M, Lal P, Donaton M, Zhang L, Hudis C (2006). An estrogen receptor-negative breast cancer subset characterized by a hormonally regulated transcriptional program and response to androgen. Oncogene.

[65] Farmer P, Bonnefoi H, Becette V, Tubiana-Hulin M, Fumoleau P, Larsimont D (2005). Identification of molecular apocrine breast tumours by microarray analysis. Oncogene.

[66] Baniwal SK, Chimge NO, Jordan VC, Tripathy D, Frenkel B (2014). Prolactin-induced protein (PIP) regulates proliferation of luminal A type breast cancer cells in an estrogen-independent manner. PLoS ONE.

[67] Bleach R, McIlroy M (2018). The divergent function of androgen receptor in breast cancer; analysis of steroid mediators and tumor intracrinology. Front Endocrinol.

[68] Sasano H, Suzuki T, Miki Y, Moriya T (2008). Intracrinology of estrogens and androgens in breast carcinoma. J Steroid Biochem Mol Biol.

[69] Panet-Raymond V, Gottlieb B, Beitel LK, Pinsky L, Trifiro MA (2000). Interactions between androgen and estrogen receptors and the effects on their transactivational properties. Mol Cell Endocrinol.

[70] Peters AA, Buchanan G, Ricciardelli C, Bianco-Miotto T, Centenera MM, Harris JM (2009). Androgen receptor inhibits estrogen receptor-alpha activity and is prognostic in breast cancer. Can Res.

[71] Poulin R, Simard J, Labrie C, Petitclerc L, Dumont M, Lagace L (1989). Down-regulation of estrogen receptors by androgens in the ZR-75-1 human breast cancer cell line. Endocrinology.

[72] Rao AR, Motiwala HG, Karim OM (2008). The discovery of prostate-specific antigen. BJU Int.

[73] Mannello F, Gazzanelli G (2001). Prostate-specific antigen (PSA/hK3): a further player in the field of breast cancer diagnostics?. Breast Cancer Res BCR.

[74] Black MH, Diamandis EP (2000). The diagnostic and prognostic utility of prostate-specific antigen for diseases of the breast. Breast Cancer Res Treat.

[75] Hautmann S, Huland E, Grupp C, Haese A, Huland H (2000). Super-sensitive prostate-specific antigen (PSA) in serum of women with benign breast disease or breast cancer. Anticancer Res.

[76] Bundred NJ, Miller WR, Walker RA (1987). An immunohistochemical study of the tissue distribution of the breast cyst fluid protein, zinc alpha 2 glycoprotein. Histopathology.

[77] Diez-Itza I, Sanchez LM, Allende MT, Vizoso F, Ruibal A, Lopez-Otin C (1993). Zn-alpha 2-glycoprotein levels in breast cancer cytosols and correlation with clinical, histological and biochemical parameters. Eur J Cancer (Oxford, England: 1990).

[78] Freije JP, Fueyo A, Uria J, Lopez-Otin C (1991). Human Zn-alpha 2-glycoprotein cDNA cloning and expression analysis in benign and malignant breast tissues. FEBS Lett.

[79] Hassan MI, Waheed A, Yadav S, Singh TP, Ahmad F (2008). Zinc alpha 2-glycoprotein: a multidisciplinary protein. Mol Cancer Res MCR.

[80] Fiel MI, Cernaianu G, Burstein DE, Batheja N (1996). Value of GCDFP-15 (BRST-2) as a specific immunocytochemical marker for breast carcinoma in cytologic specimens. Acta Cytol.

[81] Le Doussal V, Zangerle PF, Collette J, Spyratos F, Hacene K, Briere M (1985). Immunohistochemistry of a component protein of the breast cystic disease fluid with mol. wt 15,000. Eur J Cancer Clin Oncol.

[82] Murphy LC, Lee-Wing M, Goldenberg GJ, Shiu RP (1987). Expression of the gene encoding a prolactin-inducible protein by human breast cancers in vivo: correlation with steroid receptor status. Can Res.

[83] Wick MR, Lillemoe TJ, Copland GT, Swanson PE, Manivel JC, Kiang DT (1989). Gross cystic disease fluid protein-15 as a marker for breast cancer: immunohistochemical analysis of 690 human neoplasms and comparison with alpha-lactalbumin. Hum Pathol.

[84] Gangadharan A, Nyirenda T, Patel K, Jaimes-Delgadillo N, Coletta D, Tanaka T (2018). Prolactin Induced Protein (PIP) is a potential biomarker for early stage and malignant breast cancer. Breast (Edinburgh, Scotland).

[85] Haagensen DE, Mazoujian G, Dilley WG, Pedersen CE, Kister SJ, Wells SA (1979). Breast gross cystic disease fluid analysis. I. Isolation and radioimmunoassay for a major component protein. J Natl Cancer Inst.

[86] Harbeck N, Penault-Llorca F, Cortes J, Gnant M, Houssami N, Poortmans P (2019). Breast cancer. Nat Rev Dis Primers.

[87] Abreu M, Cabezas-Sainz P, Pereira-Veiga T, Falo C, Abalo A, Morilla I (2020). Looking for a better characterization of triple-negative breast cancer by means of circulating tumor cells. J Clin Med.

[88] Krawczyk N, Neubacher M, Meier-Stiegen F, Neubauer H, Niederacher D, Ruckhäberle E (2019). Determination of the androgen receptor status of circulating tumour cells in metastatic breast cancer patients. BMC Cancer.

[89] de Kruijff IE, Sieuwerts AM, Onstenk W, Jager A, Hamberg P, de Jongh FE (2019). Androgen receptor expression in circulating tumor cells of patients with metastatic breast cancer. Int J Cancer.

[90] Aceto N, Bardia A, Wittner BS, Donaldson MC, O'Keefe R, Engstrom A (2018). AR expression in breast cancer CTCs associates with bone metastases. Mol Cancer Res MCR.

[91] Keup C, Benyaa K, Hauch S, Sprenger-Haussels M, Tewes M, Mach P (2020). Targeted deep sequencing revealed variants in cell-free DNA of hormone receptor-positive metastatic breast cancer patients. Cell Mol Life Sci CMLS.

[92] Trigunaite A, Dimo J, Jorgensen TN (2015). Suppressive effects of androgens on the immune system. Cell Immunol.

[93] Hassan MI, Waheed A, Yadav S, Singh TP, Ahmad F (2009). Prolactin inducible protein in cancer, fertility and immunoregulation: structure, function and its clinical implications. Cell Mol Life Sci CMLS.

[94] Hassan MI, Bilgrami S, Kumar V, Singh N, Yadav S, Kaur P (2008). Crystal structure of the novel complex formed between zinc alpha2-glycoprotein (ZAG) and prolactin-inducible protein (PIP) from human seminal plasma. J Mol Biol.

