# Supplementary material for: Secreted indicators of androgen receptor activity in breast cancer pre-clinical models
Source: Breast Cancer Res. 2021 Nov 4;23:102. doi: 10.1186/s13058-021-01478-9 (PMC8567567; doi:10.1186/s13058-021-01478-9)
Supplement: Supplementary file 1 — Additional file 1: Fig. S1. Western blot for AR and FKBP5 in nine breast cancer cell lines. Protein extracts from whole cell lysates were examined by western blot after 4-days cultured with vehicle control, DHT (10 nM), DHT (10 nM) plus Enza (20 μM) or Enza (20 μM). [file 13058_2021_1478_MOESM1_ESM.pptx]

## Slide 1
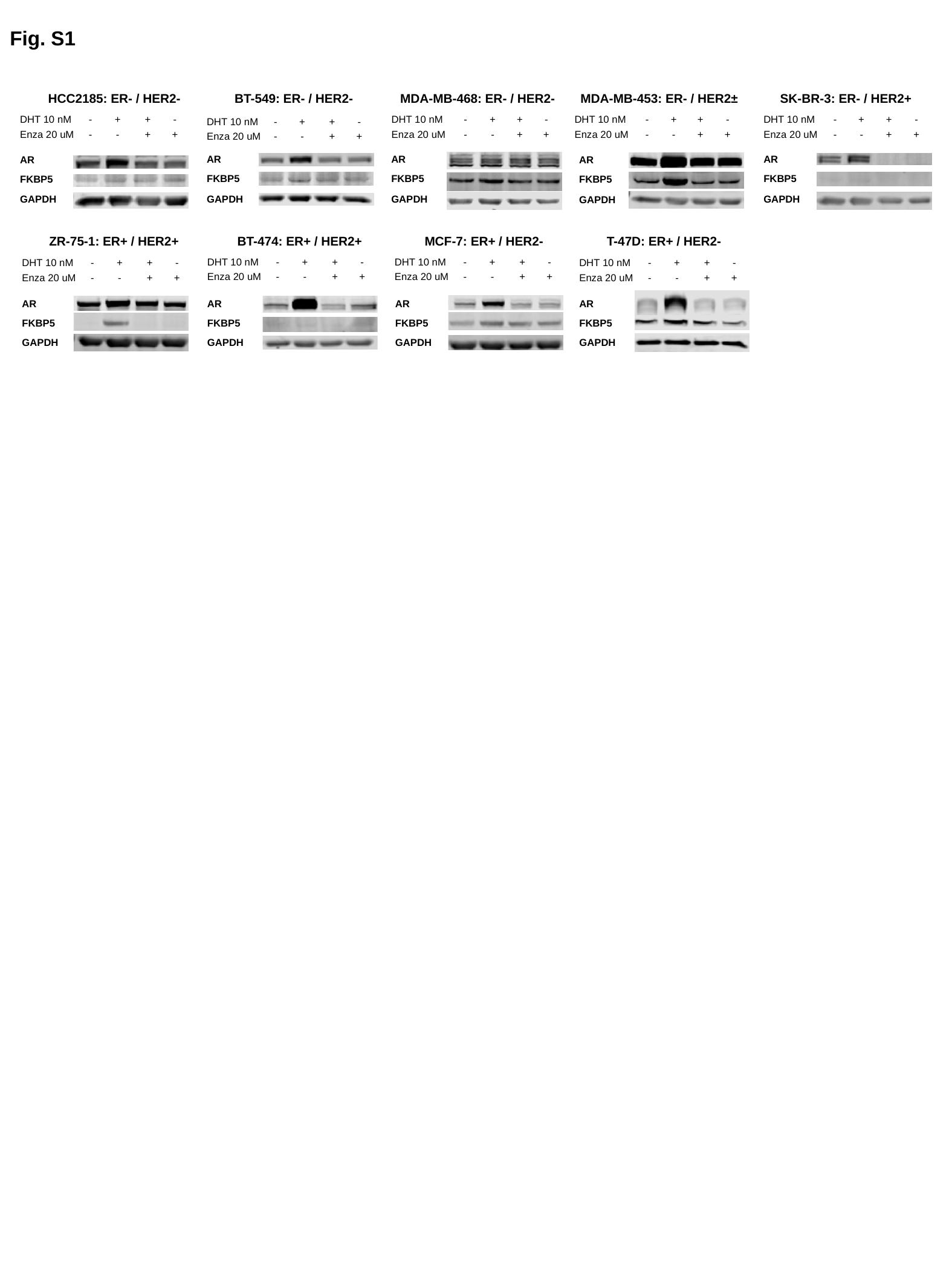

Fig. S1
HCC2185: ER- / HER2-
BT-549: ER- / HER2-
MDA-MB-468: ER- / HER2-
MDA-MB-453: ER- / HER2±
SK-BR-3: ER- / HER2+
| DHT 10 nM | - | + | + | - |
| --- | --- | --- | --- | --- |
| Enza 20 uM | - | - | + | + |
| DHT 10 nM | - | + | + | - |
| --- | --- | --- | --- | --- |
| Enza 20 uM | - | - | + | + |
| DHT 10 nM | - | + | + | - |
| --- | --- | --- | --- | --- |
| Enza 20 uM | - | - | + | + |
| DHT 10 nM | - | + | + | - |
| --- | --- | --- | --- | --- |
| Enza 20 uM | - | - | + | + |
| DHT 10 nM | - | + | + | - |
| --- | --- | --- | --- | --- |
| Enza 20 uM | - | - | + | + |
AR
AR
AR
AR
AR
FKBP5
FKBP5
FKBP5
FKBP5
FKBP5
GAPDH
GAPDH
GAPDH
GAPDH
GAPDH
ZR-75-1: ER+ / HER2+
BT-474: ER+ / HER2+
MCF-7: ER+ / HER2-
T-47D: ER+ / HER2-
| DHT 10 nM | - | + | + | - |
| --- | --- | --- | --- | --- |
| Enza 20 uM | - | - | + | + |
| DHT 10 nM | - | + | + | - |
| --- | --- | --- | --- | --- |
| Enza 20 uM | - | - | + | + |
| DHT 10 nM | - | + | + | - |
| --- | --- | --- | --- | --- |
| Enza 20 uM | - | - | + | + |
| DHT 10 nM | - | + | + | - |
| --- | --- | --- | --- | --- |
| Enza 20 uM | - | - | + | + |
AR
AR
AR
AR
FKBP5
FKBP5
FKBP5
FKBP5
GAPDH
GAPDH
GAPDH
GAPDH
