# Supplementary material for: Secreted indicators of androgen receptor activity in breast cancer pre-clinical models
Source: Breast Cancer Res. 2021 Nov 4;23:102. doi: 10.1186/s13058-021-01478-9 (PMC8567567; doi:10.1186/s13058-021-01478-9)
Supplement: Supplementary file 2 — Additional file 2: Fig. S2. AR-expressing breast cancer cells respond to Enza regardless of subtype or proliferative response to DHT. a Cell viability was assessed by crystal violet assay after 4-days cultured in full serum media with increasing concentrations of Enza. b Shown are Enza IC50 (μM) values summarized by tumor subtype. Table indicates subtype of the cell lines and corresponding Enza IC50 (μM) values. c Cell viability was assessed by crystal violet assay after 4-days cultured in hormone-depleted medium supplemented with increasing concentrations of DHT. d Shown are Enza IC50 (μM) values summarized by proliferative response to DHT. Table shows the proliferative response to DHT and Enza IC50 (μM) values for each cell line. e Expression levels of proteins relative to GAPDH were determined by western blot. Scatter plots examining correlations between protein expression and Enza IC50 levels. Regression lines are indicative of the overall correlation. [file 13058_2021_1478_MOESM2_ESM.pptx]

## Slide 1
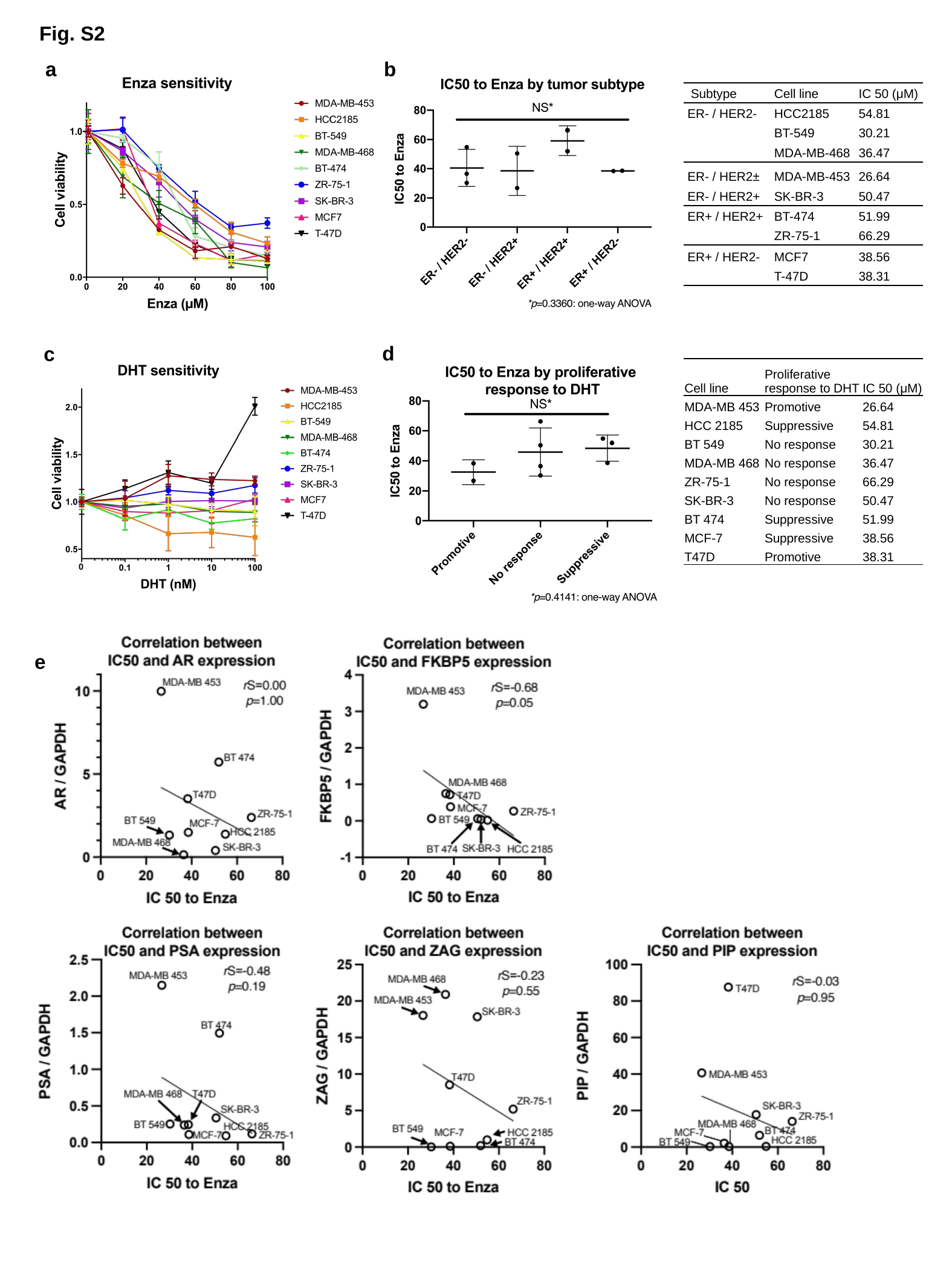

Fig. S2
a
b
| Subtype | Cell line | IC 50 (μM) |
| --- | --- | --- |
| ER- / HER2- | HCC2185 | 54.81 |
| | BT-549 | 30.21 |
| | MDA-MB-468 | 36.47 |
| ER- / HER2± | MDA-MB-453 | 26.64 |
| ER- / HER2+ | SK-BR-3 | 50.47 |
| ER+ / HER2+ | BT-474 | 51.99 |
| | ZR-75-1 | 66.29 |
| ER+ / HER2- | MCF7 | 38.56 |
| | T-47D | 38.31 |
d
c
| Cell line | Proliferative response to DHT | IC 50 (μM) |
| --- | --- | --- |
| MDA-MB 453 | Promotive | 26.64 |
| HCC 2185 | Suppressive | 54.81 |
| BT 549 | No response | 30.21 |
| MDA-MB 468 | No response | 36.47 |
| ZR-75-1 | No response | 66.29 |
| SK-BR-3 | No response | 50.47 |
| BT 474 | Suppressive | 51.99 |
| MCF-7 | Suppressive | 38.56 |
| T47D | Promotive | 38.31 |
0
e
