# Supplementary material for: Secreted indicators of androgen receptor activity in breast cancer pre-clinical models
Source: Breast Cancer Res. 2021 Nov 4;23:102. doi: 10.1186/s13058-021-01478-9 (PMC8567567; doi:10.1186/s13058-021-01478-9)
Supplement: Supplementary file 3 — Additional file 3: Fig. S3. Protein expression of PSA, ZAG and PIP in AR expressing breast cancer cell lines. Protein expression in nine cell lines were examined as shown in Fig. 3. Data for the remaining cell lines not shown in Figure 3 are shown. [file 13058_2021_1478_MOESM3_ESM.pptx]

## Slide 1
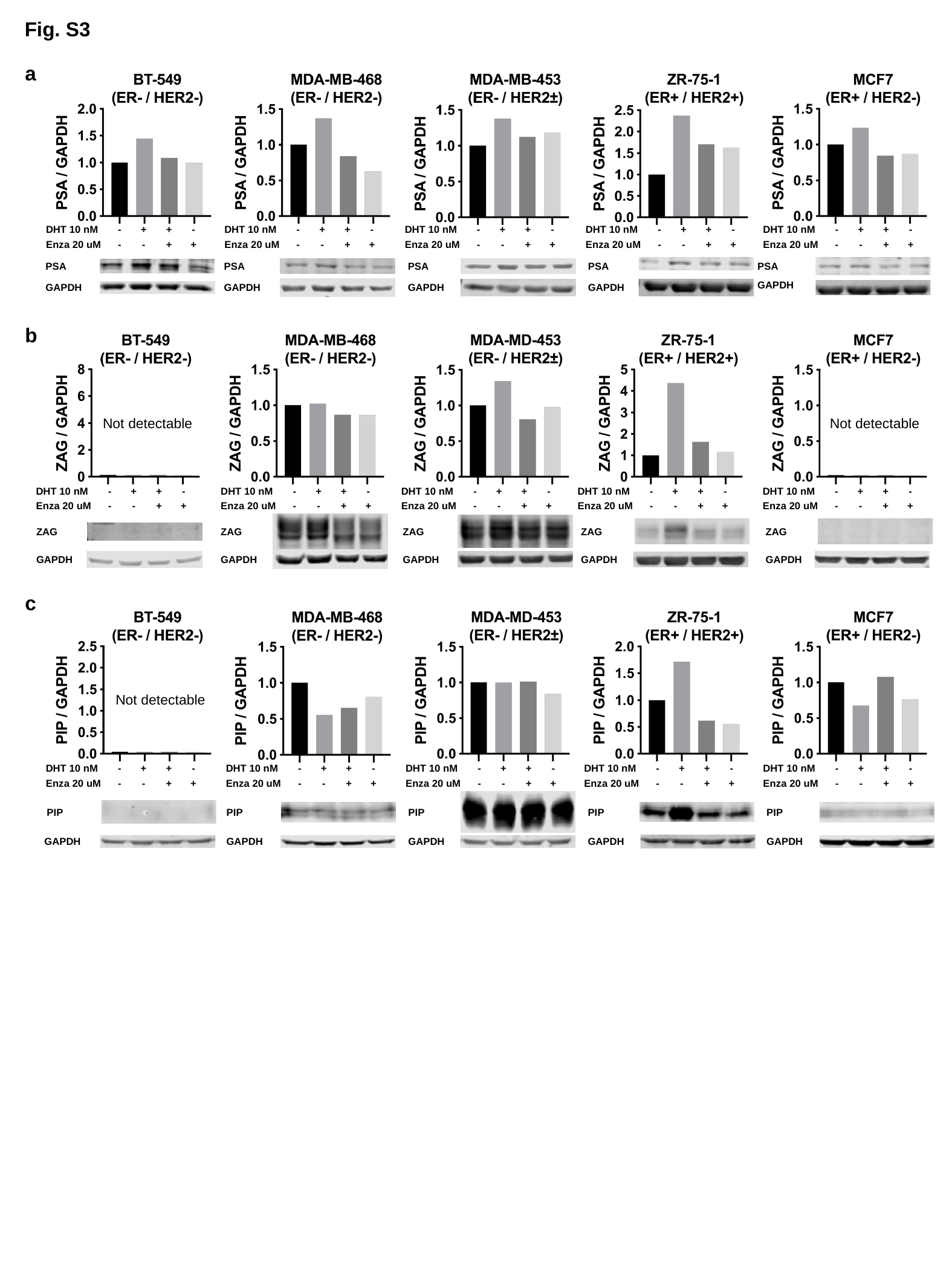

Fig. S3
a
| DHT 10 nM | - | + | + | - |
| --- | --- | --- | --- | --- |
| Enza 20 uM | - | - | + | + |
| DHT 10 nM | - | + | + | - |
| --- | --- | --- | --- | --- |
| Enza 20 uM | - | - | + | + |
| DHT 10 nM | - | + | + | - |
| --- | --- | --- | --- | --- |
| Enza 20 uM | - | - | + | + |
| DHT 10 nM | - | + | + | - |
| --- | --- | --- | --- | --- |
| Enza 20 uM | - | - | + | + |
| DHT 10 nM | - | + | + | - |
| --- | --- | --- | --- | --- |
| Enza 20 uM | - | - | + | + |
PSA
PSA
PSA
PSA
PSA
GAPDH
GAPDH
GAPDH
GAPDH
GAPDH
b
Not detectable
Not detectable
| DHT 10 nM | - | + | + | - |
| --- | --- | --- | --- | --- |
| Enza 20 uM | - | - | + | + |
| DHT 10 nM | - | + | + | - |
| --- | --- | --- | --- | --- |
| Enza 20 uM | - | - | + | + |
| DHT 10 nM | - | + | + | - |
| --- | --- | --- | --- | --- |
| Enza 20 uM | - | - | + | + |
| DHT 10 nM | - | + | + | - |
| --- | --- | --- | --- | --- |
| Enza 20 uM | - | - | + | + |
| DHT 10 nM | - | + | + | - |
| --- | --- | --- | --- | --- |
| Enza 20 uM | - | - | + | + |
ZAG
ZAG
ZAG
ZAG
ZAG
GAPDH
GAPDH
GAPDH
GAPDH
GAPDH
c
Not detectable
| DHT 10 nM | - | + | + | - |
| --- | --- | --- | --- | --- |
| Enza 20 uM | - | - | + | + |
| DHT 10 nM | - | + | + | - |
| --- | --- | --- | --- | --- |
| Enza 20 uM | - | - | + | + |
| DHT 10 nM | - | + | + | - |
| --- | --- | --- | --- | --- |
| Enza 20 uM | - | - | + | + |
| DHT 10 nM | - | + | + | - |
| --- | --- | --- | --- | --- |
| Enza 20 uM | - | - | + | + |
| DHT 10 nM | - | + | + | - |
| --- | --- | --- | --- | --- |
| Enza 20 uM | - | - | + | + |
PIP
PIP
PIP
PIP
PIP
GAPDH
GAPDH
GAPDH
GAPDH
GAPDH
