# Supplementary material for: Secreted indicators of androgen receptor activity in breast cancer pre-clinical models
Source: Breast Cancer Res. 2021 Nov 4;23:102. doi: 10.1186/s13058-021-01478-9 (PMC8567567; doi:10.1186/s13058-021-01478-9)
Supplement: Supplementary file 4 — Additional file 4: Fig. S4. Ponceau S staining of western blot conditioned media membranes. Ponceau S staining of the membranes as loading controls for western blots of conditioned medium (s, secreted proteins). [file 13058_2021_1478_MOESM4_ESM.pptx]

## Slide 1
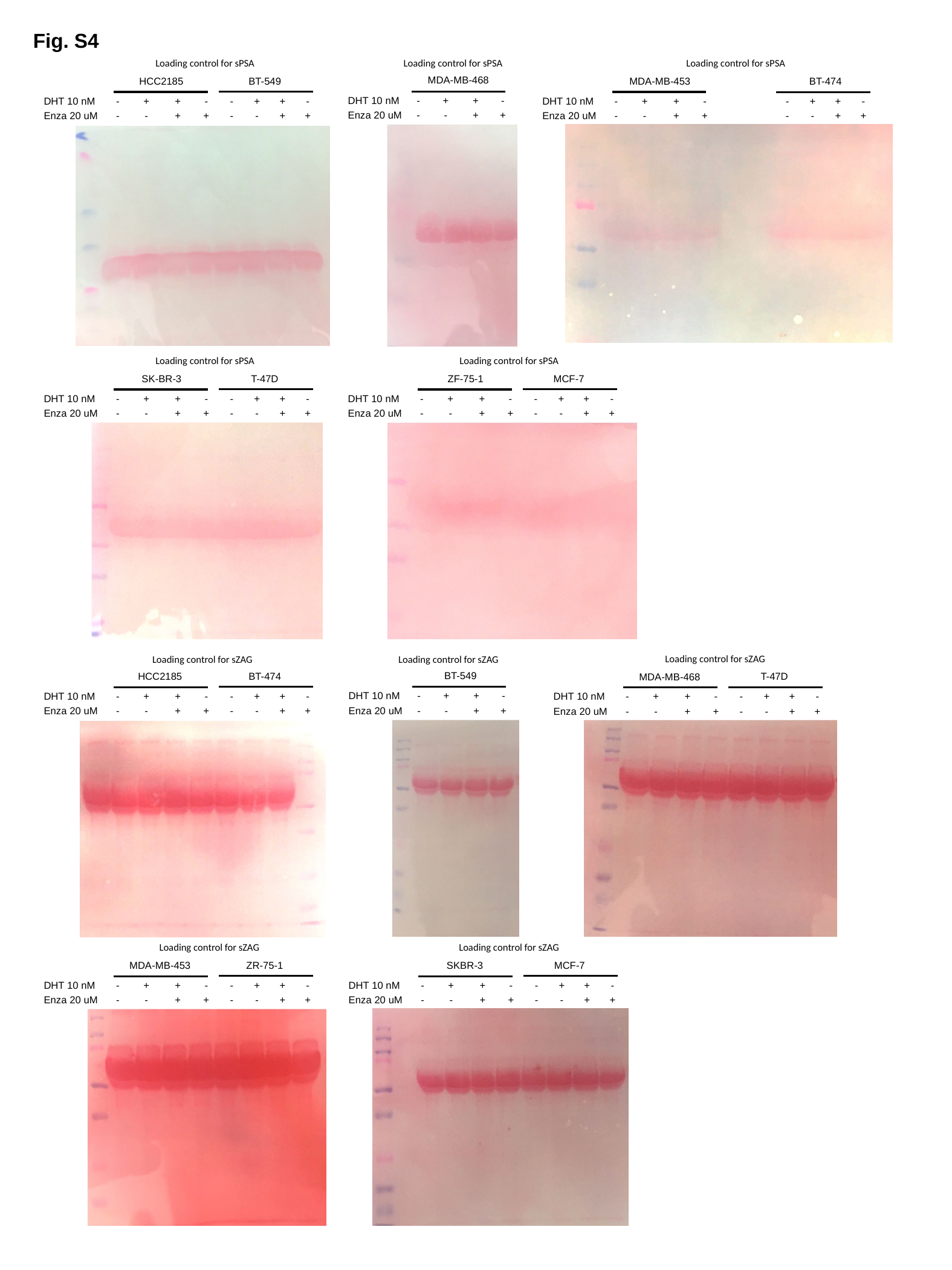

Fig. S4
Loading control for sPSA
Loading control for sPSA
Loading control for sPSA
MDA-MB-468
BT-549
MDA-MB-453
BT-474
HCC2185
| DHT 10 nM | - | + | + | - |
| --- | --- | --- | --- | --- |
| Enza 20 uM | - | - | + | + |
| DHT 10 nM | - | + | + | - | - | + | + | - |
| --- | --- | --- | --- | --- | --- | --- | --- | --- |
| Enza 20 uM | - | - | + | + | - | - | + | + |
| DHT 10 nM | - | + | + | - |
| --- | --- | --- | --- | --- |
| Enza 20 uM | - | - | + | + |
| - | + | + | - |
| --- | --- | --- | --- |
| - | - | + | + |
Loading control for sPSA
Loading control for sPSA
T-47D
MCF-7
SK-BR-3
ZF-75-1
| DHT 10 nM | - | + | + | - | - | + | + | - |
| --- | --- | --- | --- | --- | --- | --- | --- | --- |
| Enza 20 uM | - | - | + | + | - | - | + | + |
| DHT 10 nM | - | + | + | - | - | + | + | - |
| --- | --- | --- | --- | --- | --- | --- | --- | --- |
| Enza 20 uM | - | - | + | + | - | - | + | + |
Loading control for sZAG
Loading control for sZAG
Loading control for sZAG
 BT-549
BT-474
HCC2185
T-47D
MDA-MB-468
| DHT 10 nM | - | + | + | - |
| --- | --- | --- | --- | --- |
| Enza 20 uM | - | - | + | + |
| DHT 10 nM | - | + | + | - | - | + | + | - |
| --- | --- | --- | --- | --- | --- | --- | --- | --- |
| Enza 20 uM | - | - | + | + | - | - | + | + |
| DHT 10 nM | - | + | + | - | - | + | + | - |
| --- | --- | --- | --- | --- | --- | --- | --- | --- |
| Enza 20 uM | - | - | + | + | - | - | + | + |
Loading control for sZAG
Loading control for sZAG
ZR-75-1
MCF-7
MDA-MB-453
SKBR-3
| DHT 10 nM | - | + | + | - | - | + | + | - |
| --- | --- | --- | --- | --- | --- | --- | --- | --- |
| Enza 20 uM | - | - | + | + | - | - | + | + |
| DHT 10 nM | - | + | + | - | - | + | + | - |
| --- | --- | --- | --- | --- | --- | --- | --- | --- |
| Enza 20 uM | - | - | + | + | - | - | + | + |

## Slide 2
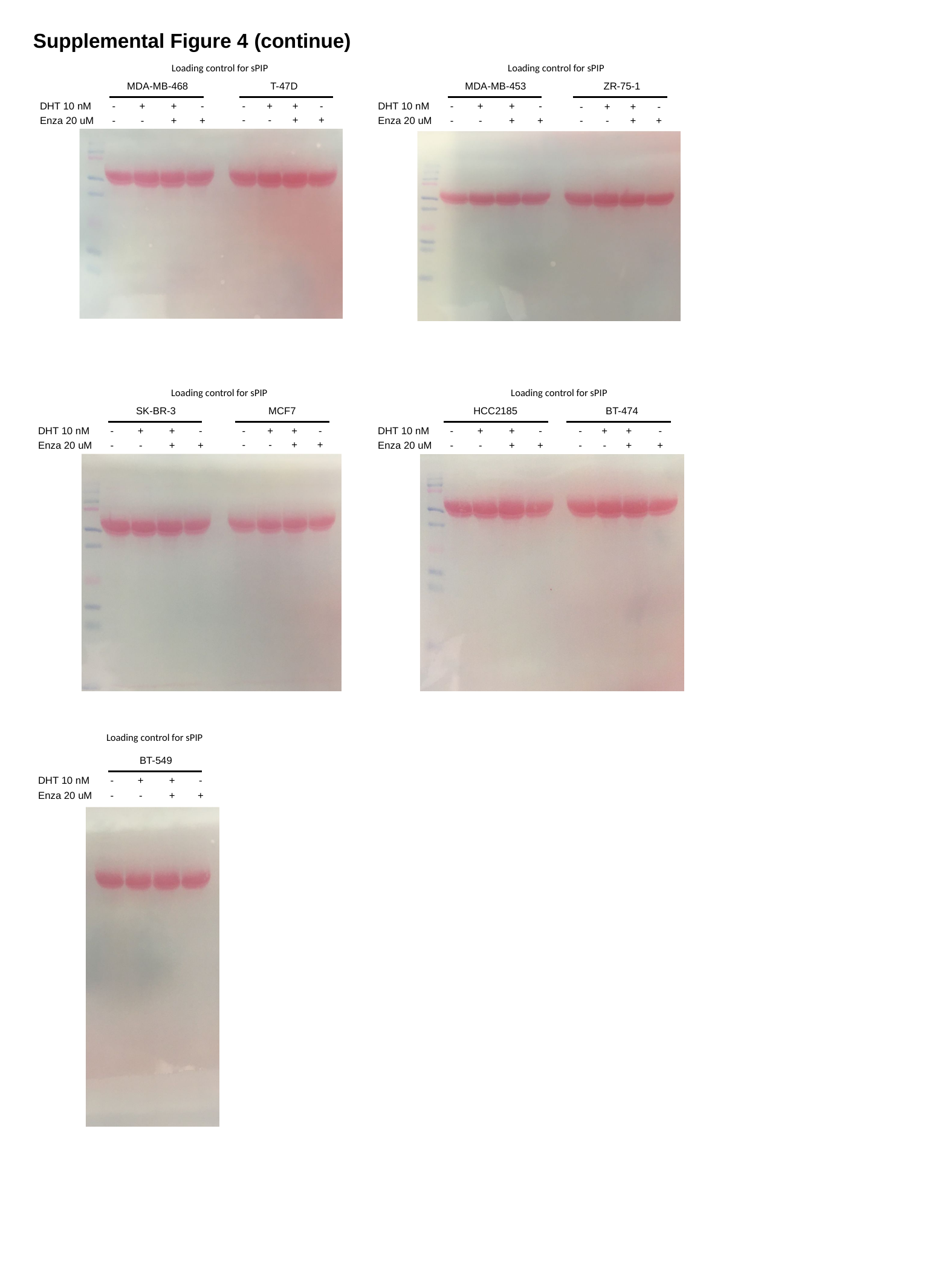

Supplemental Figure 4 (continue)
Loading control for sPIP
Loading control for sPIP
MDA-MB-468
T-47D
MDA-MB-453
ZR-75-1
| DHT 10 nM | - | + | + | - |
| --- | --- | --- | --- | --- |
| Enza 20 uM | - | - | + | + |
| - | + | + | - |
| --- | --- | --- | --- |
| - | - | + | + |
| DHT 10 nM | - | + | + | - |
| --- | --- | --- | --- | --- |
| Enza 20 uM | - | - | + | + |
| - | + | + | - |
| --- | --- | --- | --- |
| - | - | + | + |
Loading control for sPIP
Loading control for sPIP
SK-BR-3
MCF7
HCC2185
BT-474
| DHT 10 nM | - | + | + | - |
| --- | --- | --- | --- | --- |
| Enza 20 uM | - | - | + | + |
| - | + | + | - |
| --- | --- | --- | --- |
| - | - | + | + |
| DHT 10 nM | - | + | + | - |
| --- | --- | --- | --- | --- |
| Enza 20 uM | - | - | + | + |
| - | + | + | - |
| --- | --- | --- | --- |
| - | - | + | + |
Loading control for sPIP
BT-549
| DHT 10 nM | - | + | + | - |
| --- | --- | --- | --- | --- |
| Enza 20 uM | - | - | + | + |
