# Supplementary figures and images for: Secreted indicators of androgen receptor activity in breast cancer pre-clinical models
Source: Breast Cancer Res. 2021 Nov 4;23:102. doi: 10.1186/s13058-021-01478-9 (PMC8567567; doi:10.1186/s13058-021-01478-9)

## Slide 1
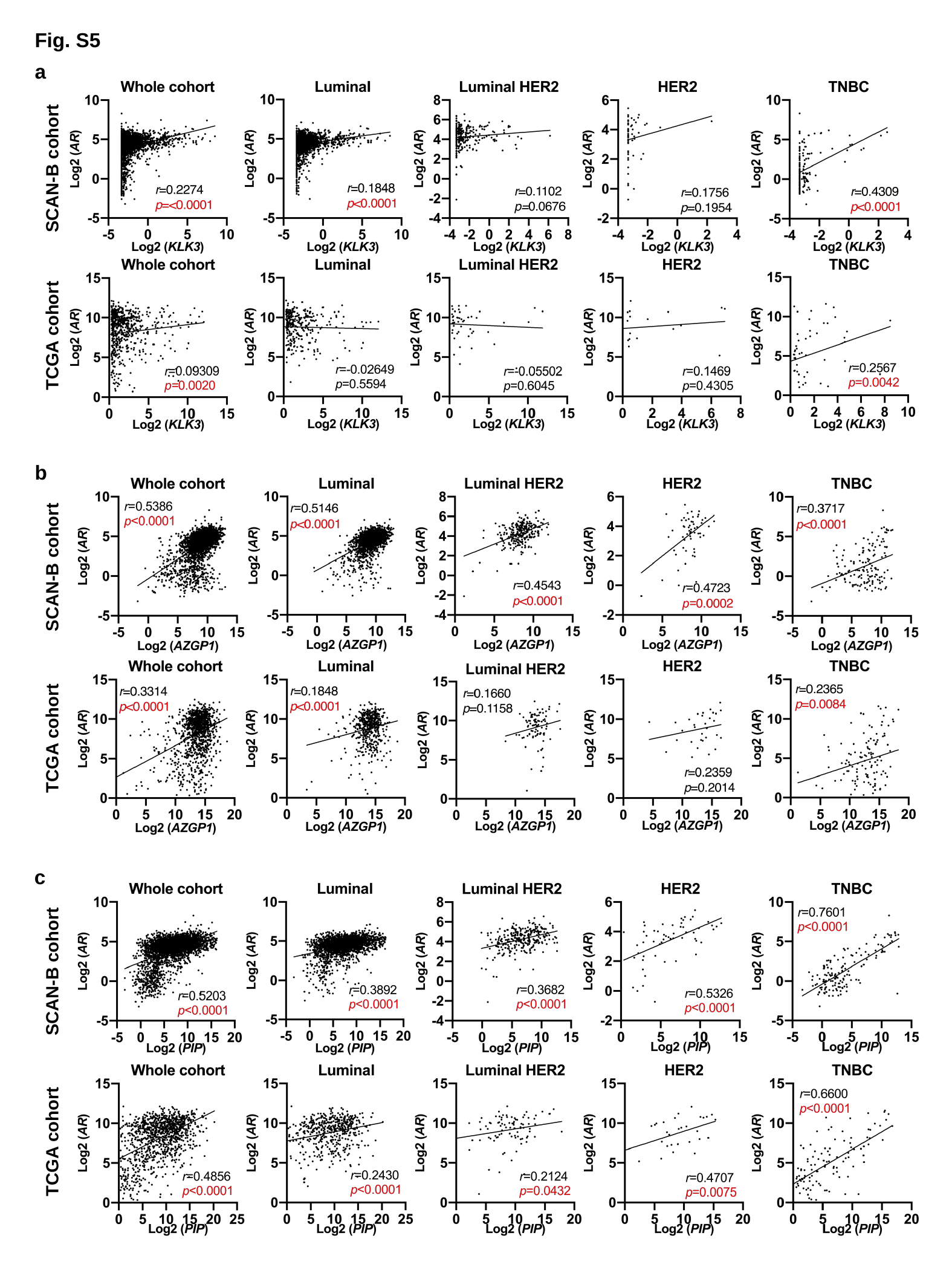

Fig. S5
a
SCAN-B cohort
TCGA cohort
b
SCAN-B cohort
TCGA cohort
c
SCAN-B cohort
TCGA cohort

Supplement: Supplementary file 5 — Additional file 5: Fig. S5. Correlation analysis of candidate gene expression and AR using gene expression profile data sets. a–c Scatter plots show the correlation between expression values for candidate genes and AR, with regression lines, Pearson’s correlation coefficients (r) and p values. [file 13058_2021_1478_MOESM5_ESM.pptx]
