# Supplementary material for: Secreted indicators of androgen receptor activity in breast cancer pre-clinical models
Source: Breast Cancer Res. 2021 Nov 4;23:102. doi: 10.1186/s13058-021-01478-9 (PMC8567567; doi:10.1186/s13058-021-01478-9)
Supplement: Supplementary file 6 — Additional file 6: Fig. S6. Association between candidate gene expression and the gene expression profile representative of tumor estrogen response. a–f The gene expression profile data from the TCGA and SCAN-B cohorts were applied to GSEA with regard to two gene sets representative of estrogen response in breast cancer cells, DUTERTRE_ESTRADIOL_RESPONSE_6HR_UP (a–c) and DUTERTRE_ESTRADIOL_ RESPONSE_24HR_UP (d–f). Enrichment plots with normal enrichment scores (NES) and p value for each cohort and subtype are shown. [file 13058_2021_1478_MOESM6_ESM.pptx]

## Slide 1
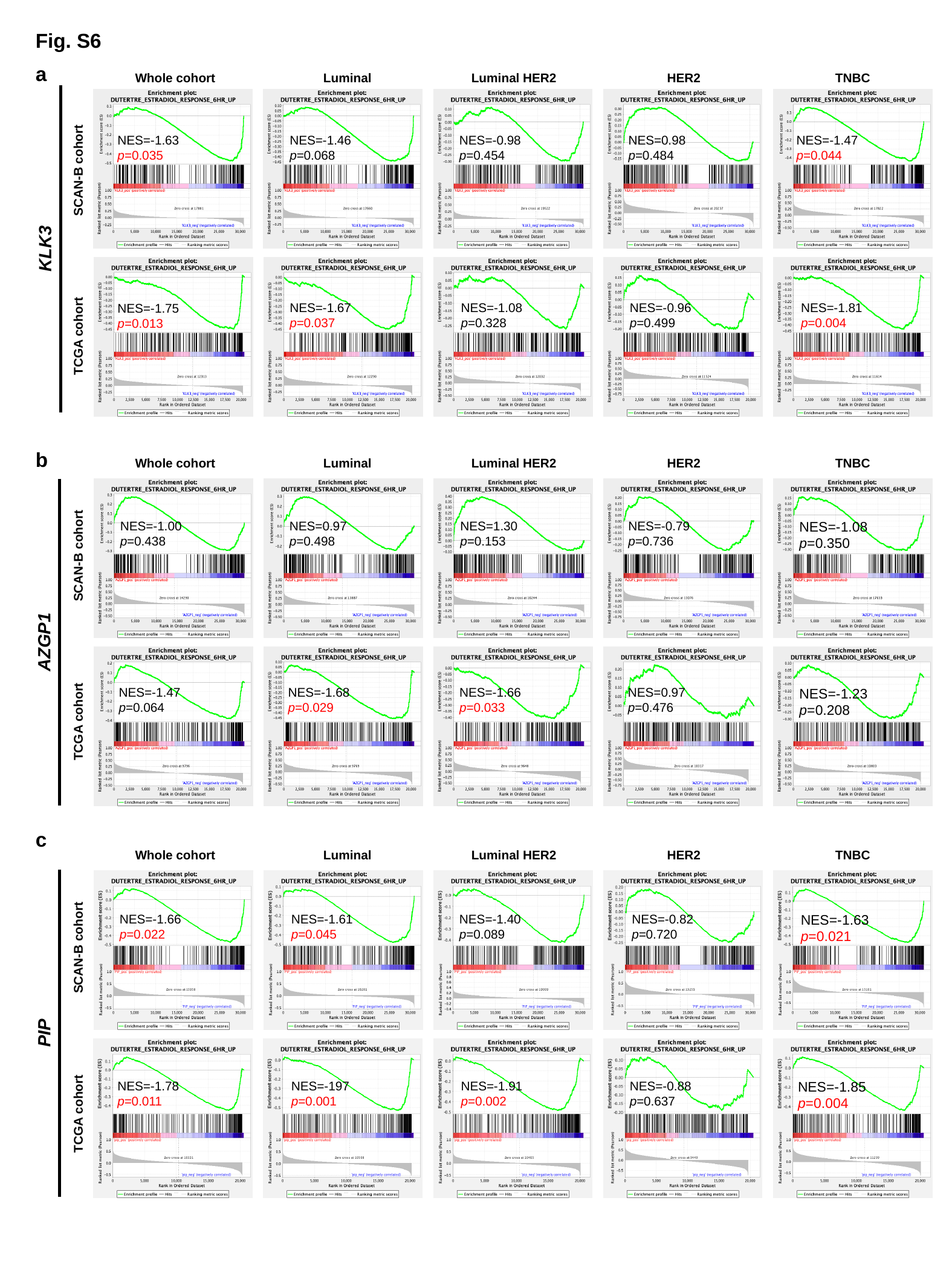

Fig. S6
a
Whole cohort
Luminal
Luminal HER2
HER2
TNBC
NES=-1.63
p=0.035
NES=-1.46
p=0.068
NES=-0.98
p=0.454
NES=0.98
p=0.484
NES=-1.47
p=0.044
SCAN-B cohort
KLK3
NES=-1.81
p=0.004
NES=-1.08
p=0.328
NES=-0.96
p=0.499
NES=-1.67
p=0.037
NES=-1.75
p=0.013
TCGA cohort
b
Whole cohort
Luminal
Luminal HER2
HER2
TNBC
NES=-1.00
p=0.438
NES=0.97
p=0.498
NES=1.30
p=0.153
NES=-0.79
p=0.736
NES=-1.08
p=0.350
SCAN-B cohort
AZGP1
NES=-1.47
p=0.064
NES=-1.68
p=0.029
NES=-1.66
p=0.033
NES=0.97
p=0.476
NES=-1.23
p=0.208
TCGA cohort
c
Whole cohort
Luminal
Luminal HER2
HER2
TNBC
NES=-1.66
p=0.022
NES=-1.61
p=0.045
NES=-1.40
p=0.089
NES=-0.82
p=0.720
NES=-1.63
p=0.021
SCAN-B cohort
PIP
NES=-1.78
p=0.011
NES=-197
p=0.001
NES=-1.91
p=0.002
NES=-0.88
p=0.637
NES=-1.85
p=0.004
TCGA cohort

## Slide 2
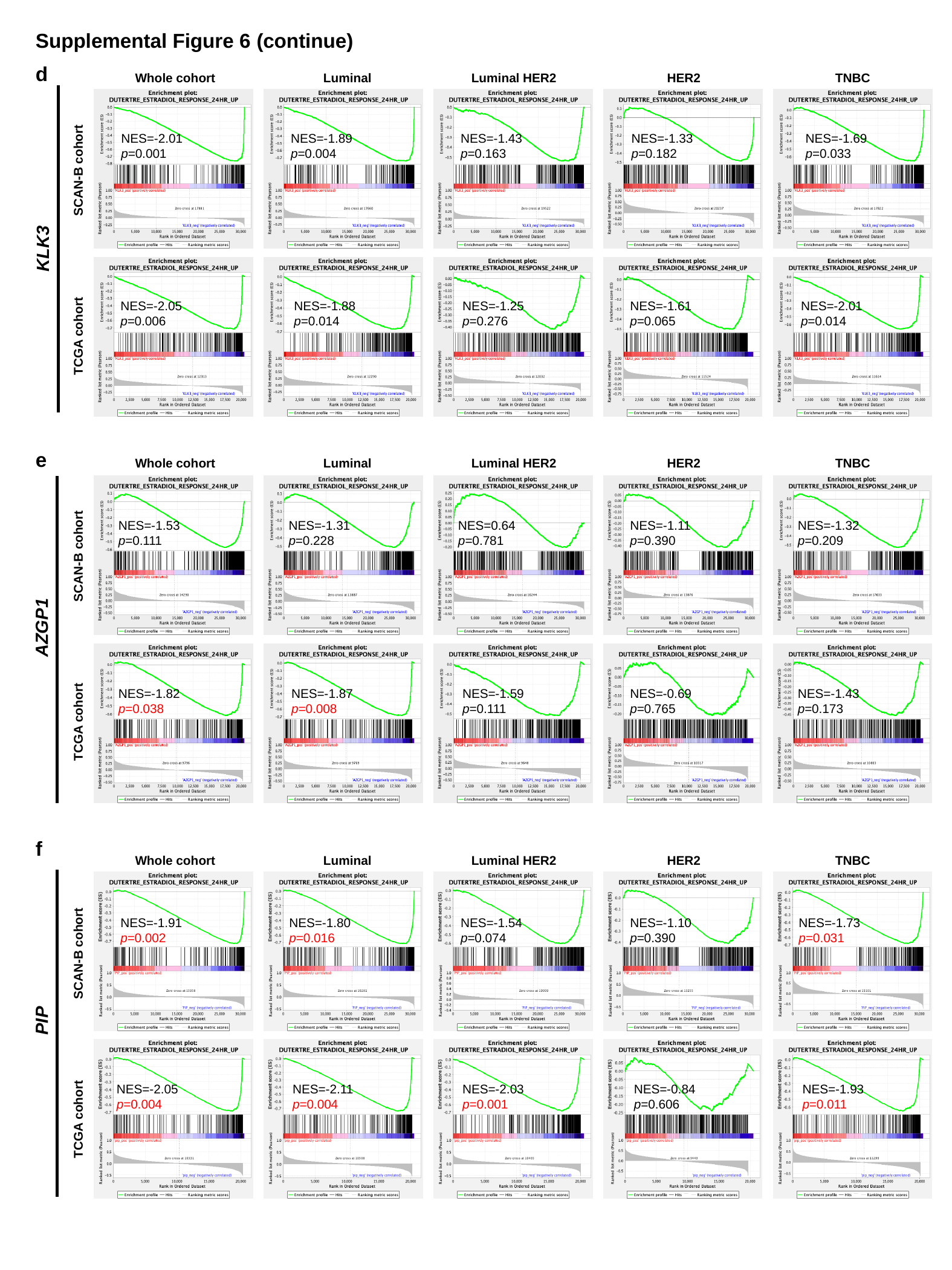

Supplemental Figure 6 (continue)
d
Whole cohort
Luminal
Luminal HER2
HER2
TNBC
NES=-2.01 p=0.001
NES=-1.89 p=0.004
NES=-1.43
p=0.163
NES=-1.33
p=0.182
NES=-1.69 p=0.033
SCAN-B cohort
KLK3
NES=-2.05
p=0.006
NES=-1.88
p=0.014
NES=-1.25
p=0.276
NES=-1.61
p=0.065
NES=-2.01
p=0.014
TCGA cohort
e
Whole cohort
Luminal
Luminal HER2
HER2
TNBC
NES=-1.53
p=0.111
NES=-1.31
p=0.228
NES=0.64
p=0.781
NES=-1.11
p=0.390
NES=-1.32
p=0.209
SCAN-B cohort
AZGP1
NES=-1.82
p=0.038
NES=-1.87
p=0.008
NES=-1.59
p=0.111
NES=-0.69
p=0.765
NES=-1.43
p=0.173
TCGA cohort
f
Whole cohort
Luminal
Luminal HER2
HER2
TNBC
NES=-1.91
p=0.002
NES=-1.80
p=0.016
NES=-1.54
p=0.074
NES=-1.10
p=0.390
NES=-1.73
p=0.031
SCAN-B cohort
PIP
NES=-2.05
p=0.004
NES=-2.11
p=0.004
NES=-2.03
p=0.001
NES=-0.84
p=0.606
NES=-1.93
p=0.011
TCGA cohort
