# Supplementary material for: Secreted indicators of androgen receptor activity in breast cancer pre-clinical models
Source: Breast Cancer Res. 2021 Nov 4;23:102. doi: 10.1186/s13058-021-01478-9 (PMC8567567; doi:10.1186/s13058-021-01478-9)
Supplement: Supplementary file 7 — Additional file 7: Fig. S7. Association between candidate gene expression and the gene expression profile representative of tumor androgen response. a–c The gene expression profile data from the TCGA and SCAN-B cohorts were applied to GSEA with regard to gene sets representative of androgen response in breast cancer cell lines, DOANE_RESPONSE_TO_ANDROGEN_UP. Enrichment plots with normal enrichment scores (NES) and p value for each cohort and subtype are shown. [file 13058_2021_1478_MOESM7_ESM.pptx]

## Slide 1
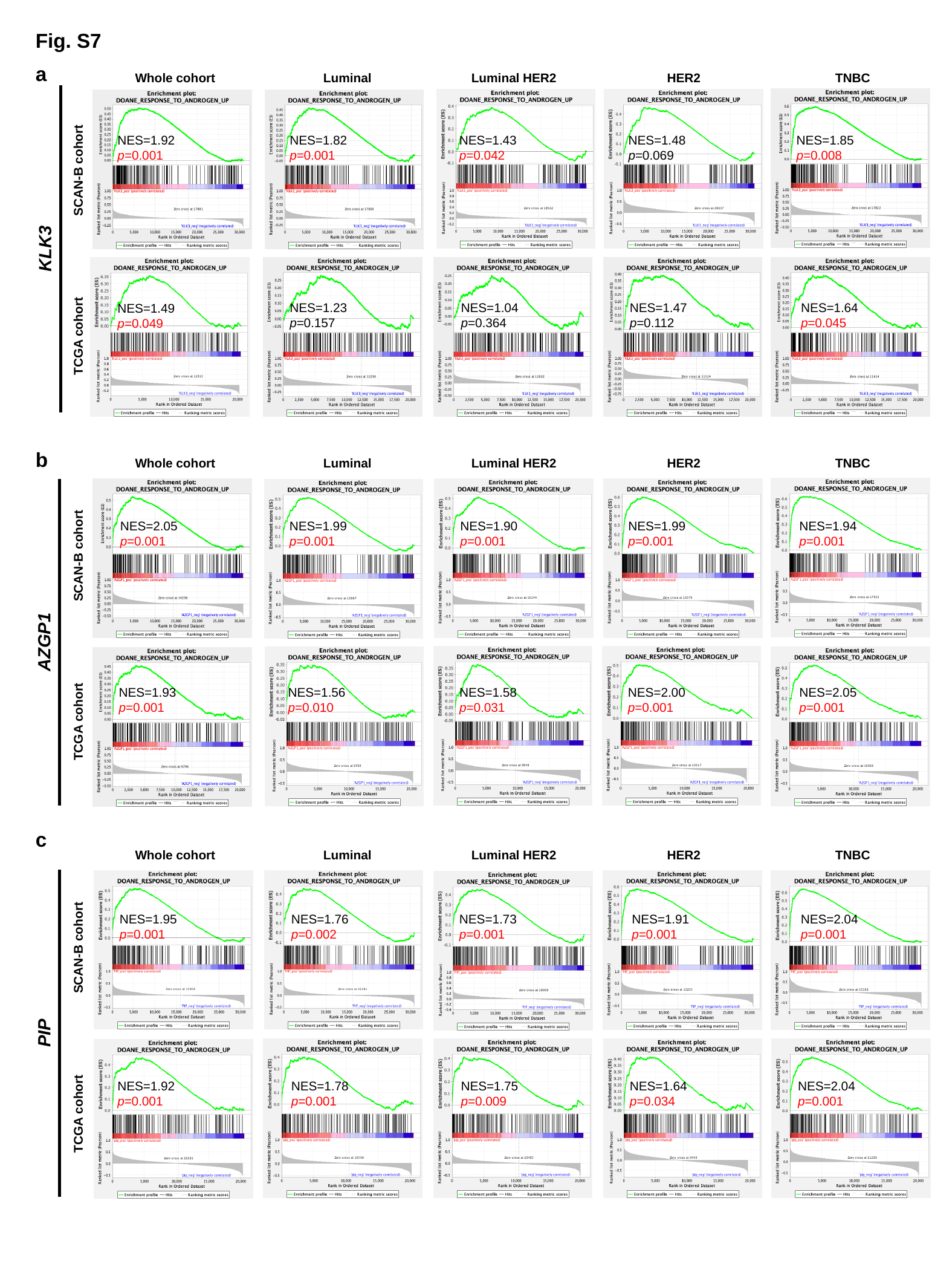

Fig. S7
a
Whole cohort
Luminal
Luminal HER2
HER2
TNBC
NES=1.92
p=0.001
NES=1.82
p=0.001
NES=1.43
p=0.042
NES=1.48
p=0.069
NES=1.85
p=0.008
SCAN-B cohort
KLK3
NES=1.64
p=0.045
NES=1.04
p=0.364
NES=1.47
p=0.112
NES=1.23
p=0.157
NES=1.49
p=0.049
TCGA cohort
b
Luminal HER2
Whole cohort
Luminal
HER2
TNBC
NES=2.05
p=0.001
NES=1.99
p=0.001
NES=1.90
p=0.001
NES=1.99
p=0.001
NES=1.94
p=0.001
SCAN-B cohort
AZGP1
NES=1.93
p=0.001
NES=1.56
p=0.010
NES=1.58
p=0.031
NES=2.00
p=0.001
NES=2.05
p=0.001
TCGA cohort
c
Whole cohort
Luminal
Luminal HER2
HER2
TNBC
NES=1.95
p=0.001
NES=1.76
p=0.002
NES=1.73
p=0.001
NES=1.91
p=0.001
NES=2.04
p=0.001
SCAN-B cohort
PIP
NES=1.92
p=0.001
NES=1.78
p=0.001
NES=1.75
p=0.009
NES=1.64
p=0.034
NES=2.04
p=0.001
TCGA cohort
