# Supplementary material for: Secreted indicators of androgen receptor activity in breast cancer pre-clinical models
Source: Breast Cancer Res. 2021 Nov 4;23:102. doi: 10.1186/s13058-021-01478-9 (PMC8567567; doi:10.1186/s13058-021-01478-9)
Supplement: Supplementary file 8 — Additional file 8: Table 1. Primer sequence used in this study. [file 13058_2021_1478_MOESM8_ESM.pptx]

## Slide 1
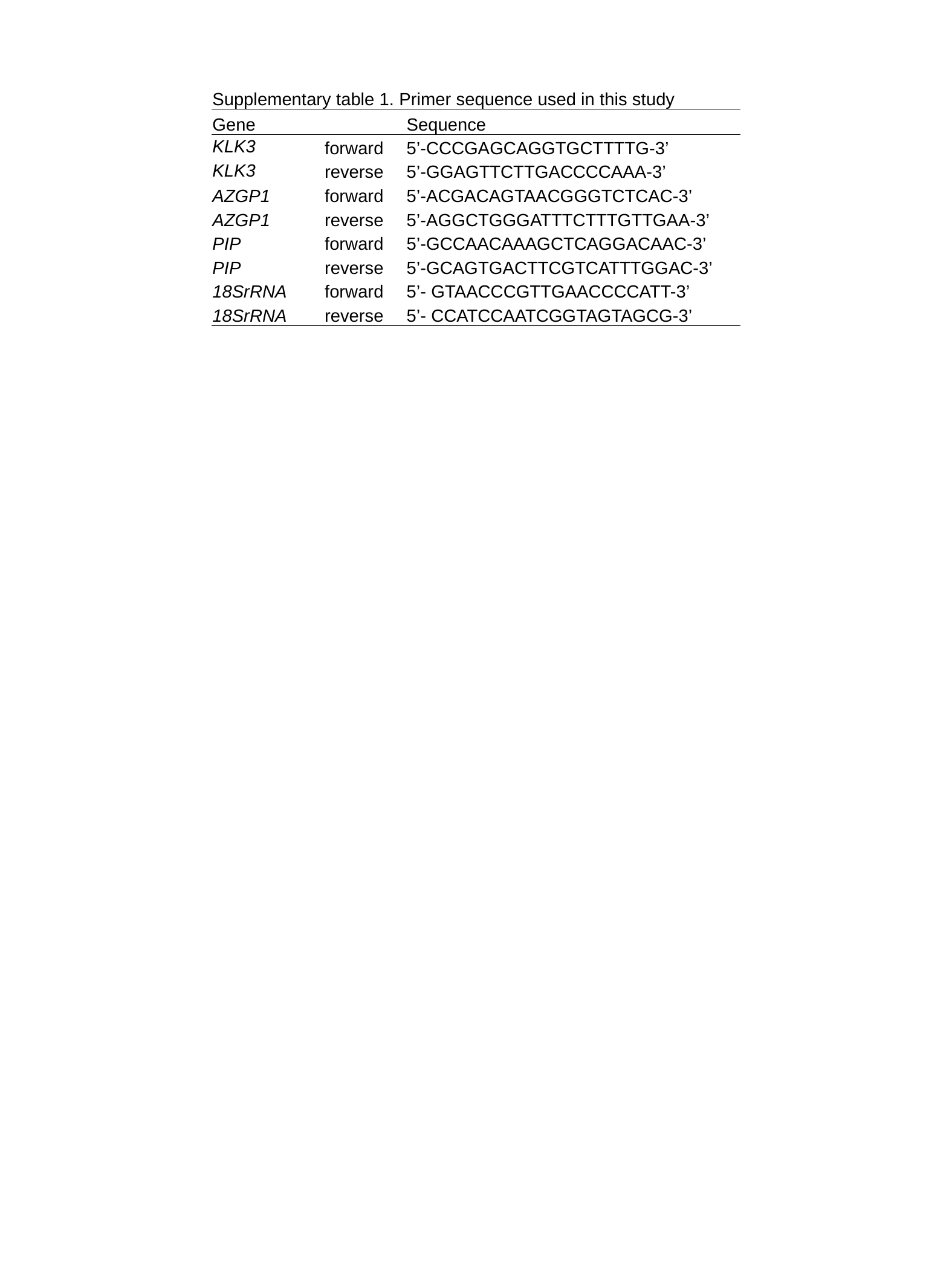

| Supplementary table 1. Primer sequence used in this study | | |
| --- | --- | --- |
| Gene | | Sequence |
| KLK3 | forward | 5’-CCCGAGCAGGTGCTTTTG-3’ |
| KLK3 | reverse | 5’-GGAGTTCTTGACCCCAAA-3’ |
| AZGP1 | forward | 5’-ACGACAGTAACGGGTCTCAC-3’ |
| AZGP1 | reverse | 5’-AGGCTGGGATTTCTTTGTTGAA-3’ |
| PIP | forward | 5’-GCCAACAAAGCTCAGGACAAC-3’ |
| PIP | reverse | 5’-GCAGTGACTTCGTCATTTGGAC-3’ |
| 18SrRNA | forward | 5’- GTAACCCGTTGAACCCCATT-3’ |
| 18SrRNA | reverse | 5’- CCATCCAATCGGTAGTAGCG-3’ |
