# Supplementary material for: Secreted indicators of androgen receptor activity in breast cancer pre-clinical models
Source: Breast Cancer Res. 2021 Nov 4;23:102. doi: 10.1186/s13058-021-01478-9 (PMC8567567; doi:10.1186/s13058-021-01478-9)
Supplement: Supplementary file 9 — Additional file 9: Table 2. Growth medium. [file 13058_2021_1478_MOESM9_ESM.pptx]

## Slide 1
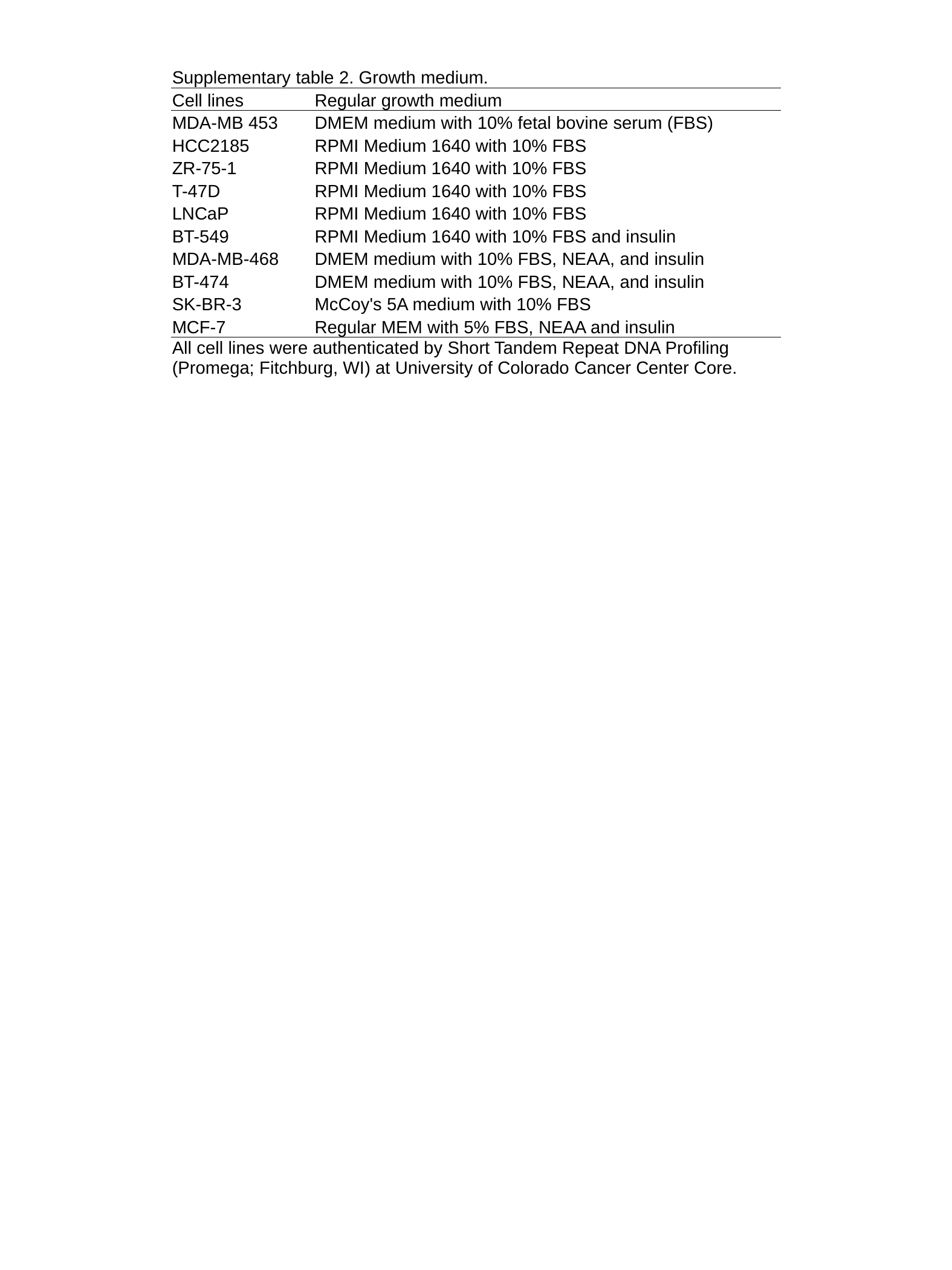

| Supplementary table 2. Growth medium. | |
| --- | --- |
| Cell lines | Regular growth medium |
| MDA-MB 453 | DMEM medium with 10% fetal bovine serum (FBS) |
| HCC2185 | RPMI Medium 1640 with 10% FBS |
| ZR-75-1 | RPMI Medium 1640 with 10% FBS |
| T-47D | RPMI Medium 1640 with 10% FBS |
| LNCaP | RPMI Medium 1640 with 10% FBS |
| BT-549 | RPMI Medium 1640 with 10% FBS and insulin |
| MDA-MB-468 | DMEM medium with 10% FBS, NEAA, and insulin |
| BT-474 | DMEM medium with 10% FBS, NEAA, and insulin |
| SK-BR-3 | McCoy's 5A medium with 10% FBS |
| MCF-7 | Regular MEM with 5% FBS, NEAA and insulin |
| All cell lines were authenticated by Short Tandem Repeat DNA Profiling (Promega; Fitchburg, WI) at University of Colorado Cancer Center Core. | |
