# Supplementary material for: Secreted indicators of androgen receptor activity in breast cancer pre-clinical models
Source: Breast Cancer Res. 2021 Nov 4;23:102. doi: 10.1186/s13058-021-01478-9 (PMC8567567; doi:10.1186/s13058-021-01478-9)
Supplement: Supplementary file 10 — Additional file 10: Table 3. Genes encoding secreted protein in breast cancer cell lines reported by Ziegler YS, et al. [file 13058_2021_1478_MOESM10_ESM.pptx]

## Slide 1
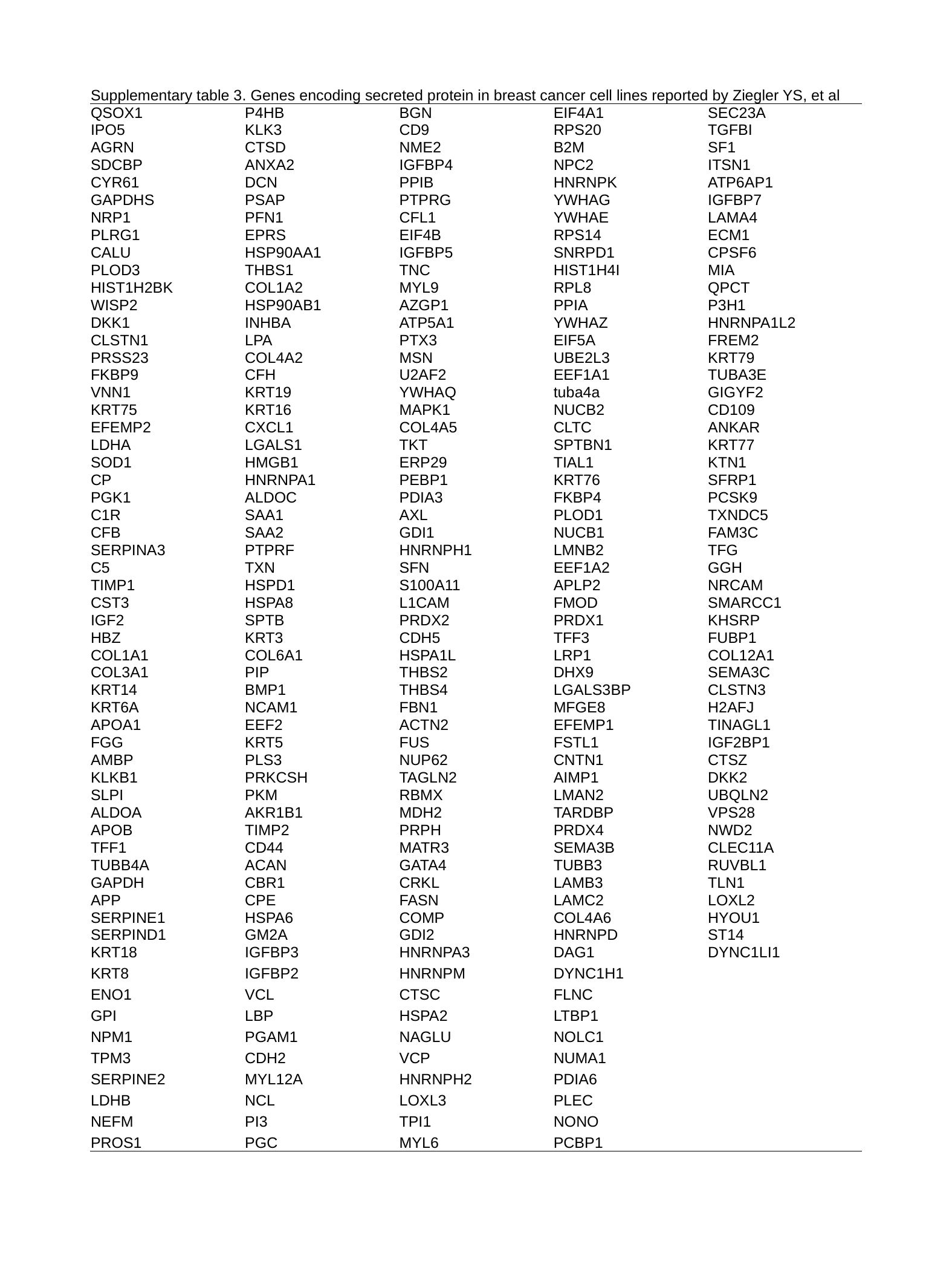

| Supplementary table 3. Genes encoding secreted protein in breast cancer cell lines reported by Ziegler YS, et al | | | | |
| --- | --- | --- | --- | --- |
| QSOX1 | P4HB | BGN | EIF4A1 | SEC23A |
| IPO5 | KLK3 | CD9 | RPS20 | TGFBI |
| AGRN | CTSD | NME2 | B2M | SF1 |
| SDCBP | ANXA2 | IGFBP4 | NPC2 | ITSN1 |
| CYR61 | DCN | PPIB | HNRNPK | ATP6AP1 |
| GAPDHS | PSAP | PTPRG | YWHAG | IGFBP7 |
| NRP1 | PFN1 | CFL1 | YWHAE | LAMA4 |
| PLRG1 | EPRS | EIF4B | RPS14 | ECM1 |
| CALU | HSP90AA1 | IGFBP5 | SNRPD1 | CPSF6 |
| PLOD3 | THBS1 | TNC | HIST1H4I | MIA |
| HIST1H2BK | COL1A2 | MYL9 | RPL8 | QPCT |
| WISP2 | HSP90AB1 | AZGP1 | PPIA | P3H1 |
| DKK1 | INHBA | ATP5A1 | YWHAZ | HNRNPA1L2 |
| CLSTN1 | LPA | PTX3 | EIF5A | FREM2 |
| PRSS23 | COL4A2 | MSN | UBE2L3 | KRT79 |
| FKBP9 | CFH | U2AF2 | EEF1A1 | TUBA3E |
| VNN1 | KRT19 | YWHAQ | tuba4a | GIGYF2 |
| KRT75 | KRT16 | MAPK1 | NUCB2 | CD109 |
| EFEMP2 | CXCL1 | COL4A5 | CLTC | ANKAR |
| LDHA | LGALS1 | TKT | SPTBN1 | KRT77 |
| SOD1 | HMGB1 | ERP29 | TIAL1 | KTN1 |
| CP | HNRNPA1 | PEBP1 | KRT76 | SFRP1 |
| PGK1 | ALDOC | PDIA3 | FKBP4 | PCSK9 |
| C1R | SAA1 | AXL | PLOD1 | TXNDC5 |
| CFB | SAA2 | GDI1 | NUCB1 | FAM3C |
| SERPINA3 | PTPRF | HNRNPH1 | LMNB2 | TFG |
| C5 | TXN | SFN | EEF1A2 | GGH |
| TIMP1 | HSPD1 | S100A11 | APLP2 | NRCAM |
| CST3 | HSPA8 | L1CAM | FMOD | SMARCC1 |
| IGF2 | SPTB | PRDX2 | PRDX1 | KHSRP |
| HBZ | KRT3 | CDH5 | TFF3 | FUBP1 |
| COL1A1 | COL6A1 | HSPA1L | LRP1 | COL12A1 |
| COL3A1 | PIP | THBS2 | DHX9 | SEMA3C |
| KRT14 | BMP1 | THBS4 | LGALS3BP | CLSTN3 |
| KRT6A | NCAM1 | FBN1 | MFGE8 | H2AFJ |
| APOA1 | EEF2 | ACTN2 | EFEMP1 | TINAGL1 |
| FGG | KRT5 | FUS | FSTL1 | IGF2BP1 |
| AMBP | PLS3 | NUP62 | CNTN1 | CTSZ |
| KLKB1 | PRKCSH | TAGLN2 | AIMP1 | DKK2 |
| SLPI | PKM | RBMX | LMAN2 | UBQLN2 |
| ALDOA | AKR1B1 | MDH2 | TARDBP | VPS28 |
| APOB | TIMP2 | PRPH | PRDX4 | NWD2 |
| TFF1 | CD44 | MATR3 | SEMA3B | CLEC11A |
| TUBB4A | ACAN | GATA4 | TUBB3 | RUVBL1 |
| GAPDH | CBR1 | CRKL | LAMB3 | TLN1 |
| APP | CPE | FASN | LAMC2 | LOXL2 |
| SERPINE1 | HSPA6 | COMP | COL4A6 | HYOU1 |
| SERPIND1 | GM2A | GDI2 | HNRNPD | ST14 |
| KRT18 | IGFBP3 | HNRNPA3 | DAG1 | DYNC1LI1 |
| KRT8 | IGFBP2 | HNRNPM | DYNC1H1 | |
| ENO1 | VCL | CTSC | FLNC | |
| GPI | LBP | HSPA2 | LTBP1 | |
| NPM1 | PGAM1 | NAGLU | NOLC1 | |
| TPM3 | CDH2 | VCP | NUMA1 | |
| SERPINE2 | MYL12A | HNRNPH2 | PDIA6 | |
| LDHB | NCL | LOXL3 | PLEC | |
| NEFM | PI3 | TPI1 | NONO | |
| PROS1 | PGC | MYL6 | PCBP1 | |
