# Supplementary material for: Secreted indicators of androgen receptor activity in breast cancer pre-clinical models
Source: Breast Cancer Res. 2021 Nov 4;23:102. doi: 10.1186/s13058-021-01478-9 (PMC8567567; doi:10.1186/s13058-021-01478-9)
Supplement: Supplementary file 11 — Additional file 11: Table 4. Comparison of proliferative response to Enza between cell lines. [file 13058_2021_1478_MOESM11_ESM.pptx]

## Slide 1
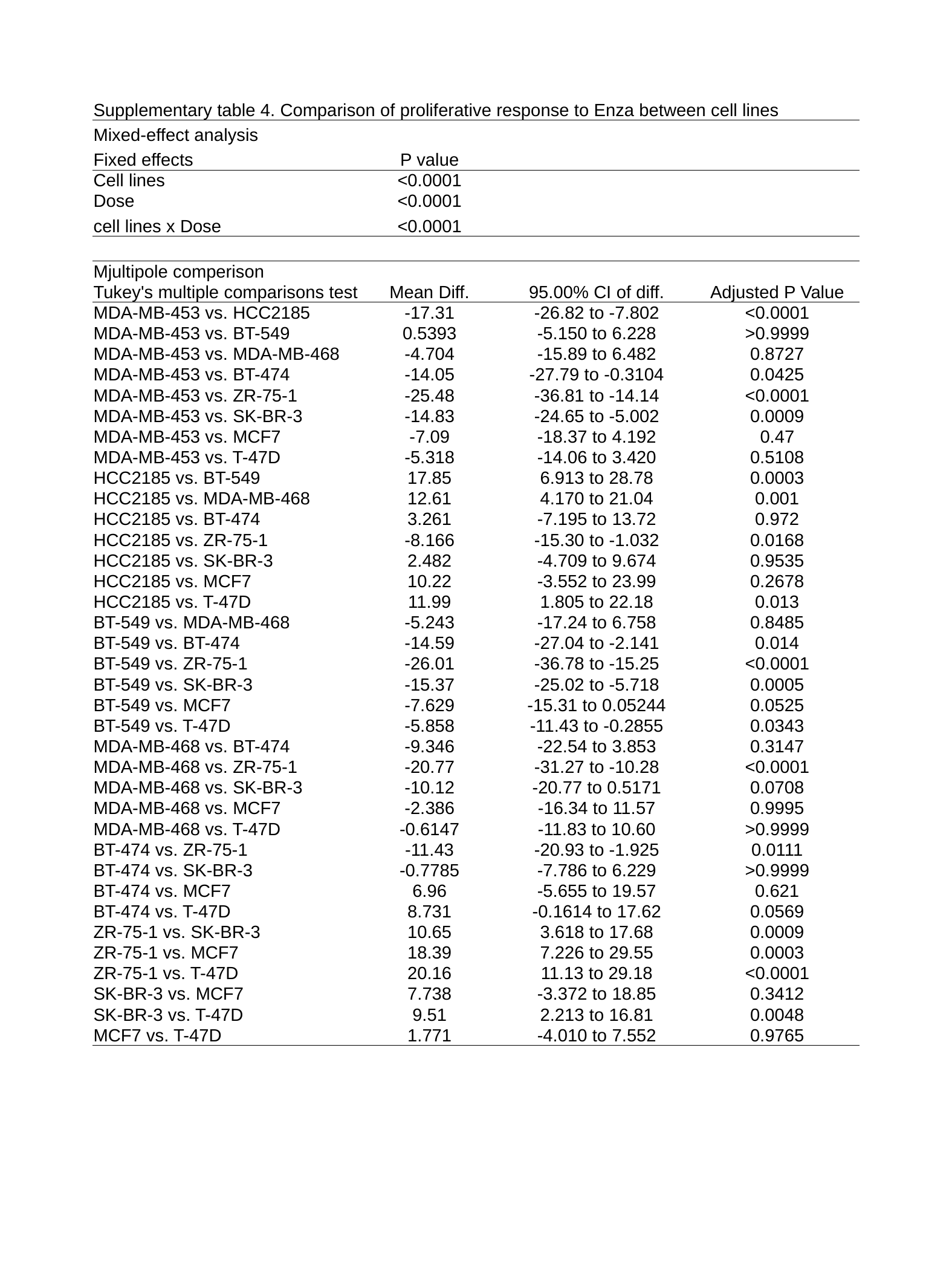

| Supplementary table 4. Comparison of proliferative response to Enza between cell lines | | | |
| --- | --- | --- | --- |
| Mixed-effect analysis | | | |
| Fixed effects | P value | | |
| Cell lines | <0.0001 | | |
| Dose | <0.0001 | | |
| cell lines x Dose | <0.0001 | | |
| | | | |
| Mjultipole comperison | | | |
| Tukey's multiple comparisons test | Mean Diff. | 95.00% CI of diff. | Adjusted P Value |
| MDA-MB-453 vs. HCC2185 | -17.31 | -26.82 to -7.802 | <0.0001 |
| MDA-MB-453 vs. BT-549 | 0.5393 | -5.150 to 6.228 | >0.9999 |
| MDA-MB-453 vs. MDA-MB-468 | -4.704 | -15.89 to 6.482 | 0.8727 |
| MDA-MB-453 vs. BT-474 | -14.05 | -27.79 to -0.3104 | 0.0425 |
| MDA-MB-453 vs. ZR-75-1 | -25.48 | -36.81 to -14.14 | <0.0001 |
| MDA-MB-453 vs. SK-BR-3 | -14.83 | -24.65 to -5.002 | 0.0009 |
| MDA-MB-453 vs. MCF7 | -7.09 | -18.37 to 4.192 | 0.47 |
| MDA-MB-453 vs. T-47D | -5.318 | -14.06 to 3.420 | 0.5108 |
| HCC2185 vs. BT-549 | 17.85 | 6.913 to 28.78 | 0.0003 |
| HCC2185 vs. MDA-MB-468 | 12.61 | 4.170 to 21.04 | 0.001 |
| HCC2185 vs. BT-474 | 3.261 | -7.195 to 13.72 | 0.972 |
| HCC2185 vs. ZR-75-1 | -8.166 | -15.30 to -1.032 | 0.0168 |
| HCC2185 vs. SK-BR-3 | 2.482 | -4.709 to 9.674 | 0.9535 |
| HCC2185 vs. MCF7 | 10.22 | -3.552 to 23.99 | 0.2678 |
| HCC2185 vs. T-47D | 11.99 | 1.805 to 22.18 | 0.013 |
| BT-549 vs. MDA-MB-468 | -5.243 | -17.24 to 6.758 | 0.8485 |
| BT-549 vs. BT-474 | -14.59 | -27.04 to -2.141 | 0.014 |
| BT-549 vs. ZR-75-1 | -26.01 | -36.78 to -15.25 | <0.0001 |
| BT-549 vs. SK-BR-3 | -15.37 | -25.02 to -5.718 | 0.0005 |
| BT-549 vs. MCF7 | -7.629 | -15.31 to 0.05244 | 0.0525 |
| BT-549 vs. T-47D | -5.858 | -11.43 to -0.2855 | 0.0343 |
| MDA-MB-468 vs. BT-474 | -9.346 | -22.54 to 3.853 | 0.3147 |
| MDA-MB-468 vs. ZR-75-1 | -20.77 | -31.27 to -10.28 | <0.0001 |
| MDA-MB-468 vs. SK-BR-3 | -10.12 | -20.77 to 0.5171 | 0.0708 |
| MDA-MB-468 vs. MCF7 | -2.386 | -16.34 to 11.57 | 0.9995 |
| MDA-MB-468 vs. T-47D | -0.6147 | -11.83 to 10.60 | >0.9999 |
| BT-474 vs. ZR-75-1 | -11.43 | -20.93 to -1.925 | 0.0111 |
| BT-474 vs. SK-BR-3 | -0.7785 | -7.786 to 6.229 | >0.9999 |
| BT-474 vs. MCF7 | 6.96 | -5.655 to 19.57 | 0.621 |
| BT-474 vs. T-47D | 8.731 | -0.1614 to 17.62 | 0.0569 |
| ZR-75-1 vs. SK-BR-3 | 10.65 | 3.618 to 17.68 | 0.0009 |
| ZR-75-1 vs. MCF7 | 18.39 | 7.226 to 29.55 | 0.0003 |
| ZR-75-1 vs. T-47D | 20.16 | 11.13 to 29.18 | <0.0001 |
| SK-BR-3 vs. MCF7 | 7.738 | -3.372 to 18.85 | 0.3412 |
| SK-BR-3 vs. T-47D | 9.51 | 2.213 to 16.81 | 0.0048 |
| MCF7 vs. T-47D | 1.771 | -4.010 to 7.552 | 0.9765 |
| | | | |
