# Supplementary material for: Secreted indicators of androgen receptor activity in breast cancer pre-clinical models
Source: Breast Cancer Res. 2021 Nov 4;23:102. doi: 10.1186/s13058-021-01478-9 (PMC8567567; doi:10.1186/s13058-021-01478-9)
Supplement: Supplementary file 12 — Additional file 12: Table 5. Comparison of proliferative response to DHT between cell lines. [file 13058_2021_1478_MOESM12_ESM.pptx]

## Slide 1
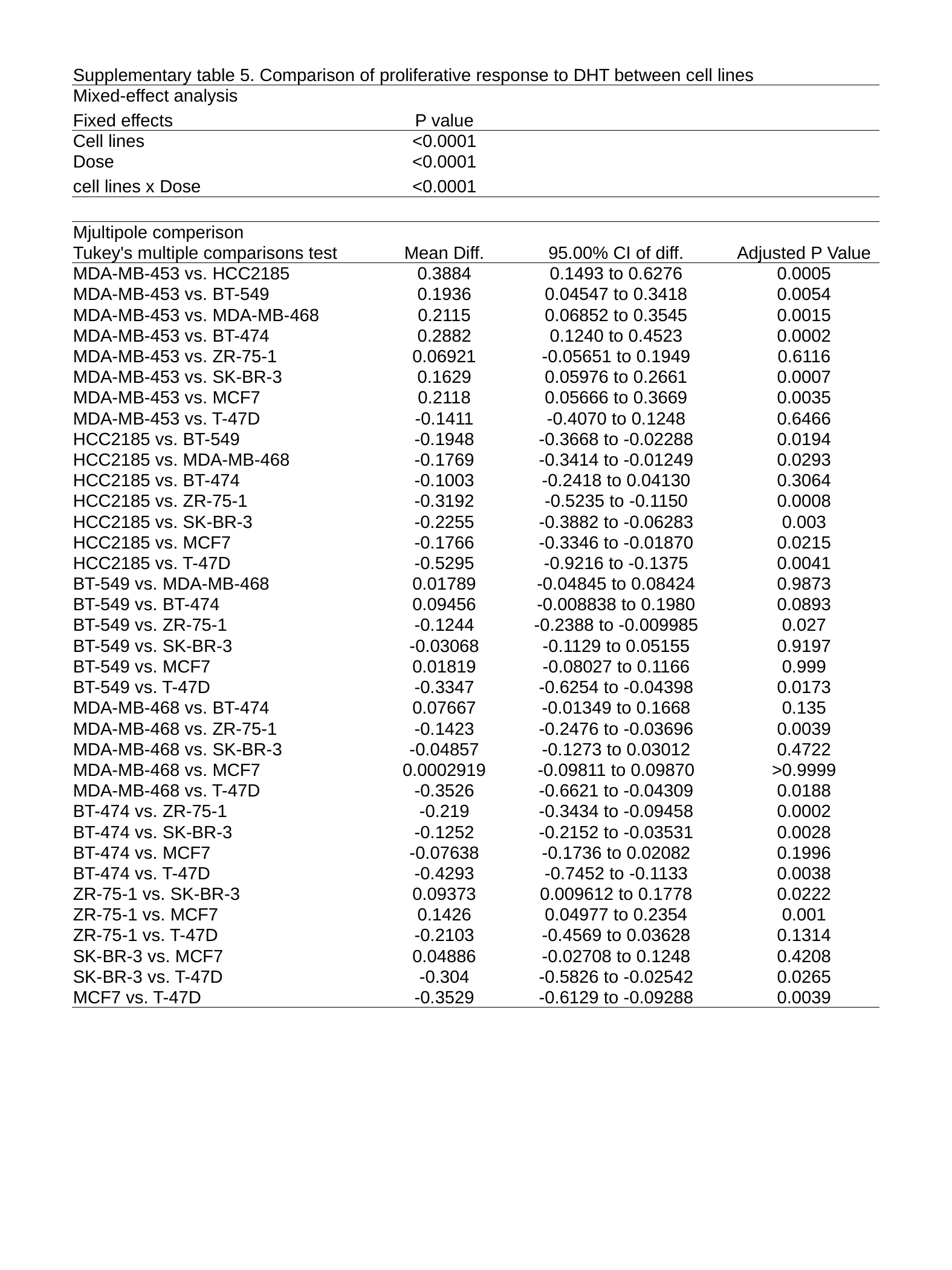

| Supplementary table 5. Comparison of proliferative response to DHT between cell lines | | | |
| --- | --- | --- | --- |
| Mixed-effect analysis | | | |
| Fixed effects | P value | | |
| Cell lines | <0.0001 | | |
| Dose | <0.0001 | | |
| cell lines x Dose | <0.0001 | | |
| | | | |
| Mjultipole comperison | | | |
| Tukey's multiple comparisons test | Mean Diff. | 95.00% CI of diff. | Adjusted P Value |
| MDA-MB-453 vs. HCC2185 | 0.3884 | 0.1493 to 0.6276 | 0.0005 |
| MDA-MB-453 vs. BT-549 | 0.1936 | 0.04547 to 0.3418 | 0.0054 |
| MDA-MB-453 vs. MDA-MB-468 | 0.2115 | 0.06852 to 0.3545 | 0.0015 |
| MDA-MB-453 vs. BT-474 | 0.2882 | 0.1240 to 0.4523 | 0.0002 |
| MDA-MB-453 vs. ZR-75-1 | 0.06921 | -0.05651 to 0.1949 | 0.6116 |
| MDA-MB-453 vs. SK-BR-3 | 0.1629 | 0.05976 to 0.2661 | 0.0007 |
| MDA-MB-453 vs. MCF7 | 0.2118 | 0.05666 to 0.3669 | 0.0035 |
| MDA-MB-453 vs. T-47D | -0.1411 | -0.4070 to 0.1248 | 0.6466 |
| HCC2185 vs. BT-549 | -0.1948 | -0.3668 to -0.02288 | 0.0194 |
| HCC2185 vs. MDA-MB-468 | -0.1769 | -0.3414 to -0.01249 | 0.0293 |
| HCC2185 vs. BT-474 | -0.1003 | -0.2418 to 0.04130 | 0.3064 |
| HCC2185 vs. ZR-75-1 | -0.3192 | -0.5235 to -0.1150 | 0.0008 |
| HCC2185 vs. SK-BR-3 | -0.2255 | -0.3882 to -0.06283 | 0.003 |
| HCC2185 vs. MCF7 | -0.1766 | -0.3346 to -0.01870 | 0.0215 |
| HCC2185 vs. T-47D | -0.5295 | -0.9216 to -0.1375 | 0.0041 |
| BT-549 vs. MDA-MB-468 | 0.01789 | -0.04845 to 0.08424 | 0.9873 |
| BT-549 vs. BT-474 | 0.09456 | -0.008838 to 0.1980 | 0.0893 |
| BT-549 vs. ZR-75-1 | -0.1244 | -0.2388 to -0.009985 | 0.027 |
| BT-549 vs. SK-BR-3 | -0.03068 | -0.1129 to 0.05155 | 0.9197 |
| BT-549 vs. MCF7 | 0.01819 | -0.08027 to 0.1166 | 0.999 |
| BT-549 vs. T-47D | -0.3347 | -0.6254 to -0.04398 | 0.0173 |
| MDA-MB-468 vs. BT-474 | 0.07667 | -0.01349 to 0.1668 | 0.135 |
| MDA-MB-468 vs. ZR-75-1 | -0.1423 | -0.2476 to -0.03696 | 0.0039 |
| MDA-MB-468 vs. SK-BR-3 | -0.04857 | -0.1273 to 0.03012 | 0.4722 |
| MDA-MB-468 vs. MCF7 | 0.0002919 | -0.09811 to 0.09870 | >0.9999 |
| MDA-MB-468 vs. T-47D | -0.3526 | -0.6621 to -0.04309 | 0.0188 |
| BT-474 vs. ZR-75-1 | -0.219 | -0.3434 to -0.09458 | 0.0002 |
| BT-474 vs. SK-BR-3 | -0.1252 | -0.2152 to -0.03531 | 0.0028 |
| BT-474 vs. MCF7 | -0.07638 | -0.1736 to 0.02082 | 0.1996 |
| BT-474 vs. T-47D | -0.4293 | -0.7452 to -0.1133 | 0.0038 |
| ZR-75-1 vs. SK-BR-3 | 0.09373 | 0.009612 to 0.1778 | 0.0222 |
| ZR-75-1 vs. MCF7 | 0.1426 | 0.04977 to 0.2354 | 0.001 |
| ZR-75-1 vs. T-47D | -0.2103 | -0.4569 to 0.03628 | 0.1314 |
| SK-BR-3 vs. MCF7 | 0.04886 | -0.02708 to 0.1248 | 0.4208 |
| SK-BR-3 vs. T-47D | -0.304 | -0.5826 to -0.02542 | 0.0265 |
| MCF7 vs. T-47D | -0.3529 | -0.6129 to -0.09288 | 0.0039 |
