# Supplementary material for: Secreted indicators of androgen receptor activity in breast cancer pre-clinical models
Source: Breast Cancer Res. 2021 Nov 4;23:102. doi: 10.1186/s13058-021-01478-9 (PMC8567567; doi:10.1186/s13058-021-01478-9)
Supplement: Supplementary file 13 — Additional file 13: Table 6. Comparison of proliferative response to different dose of DHT in same cell lines. [file 13058_2021_1478_MOESM13_ESM.pptx]

## Slide 1
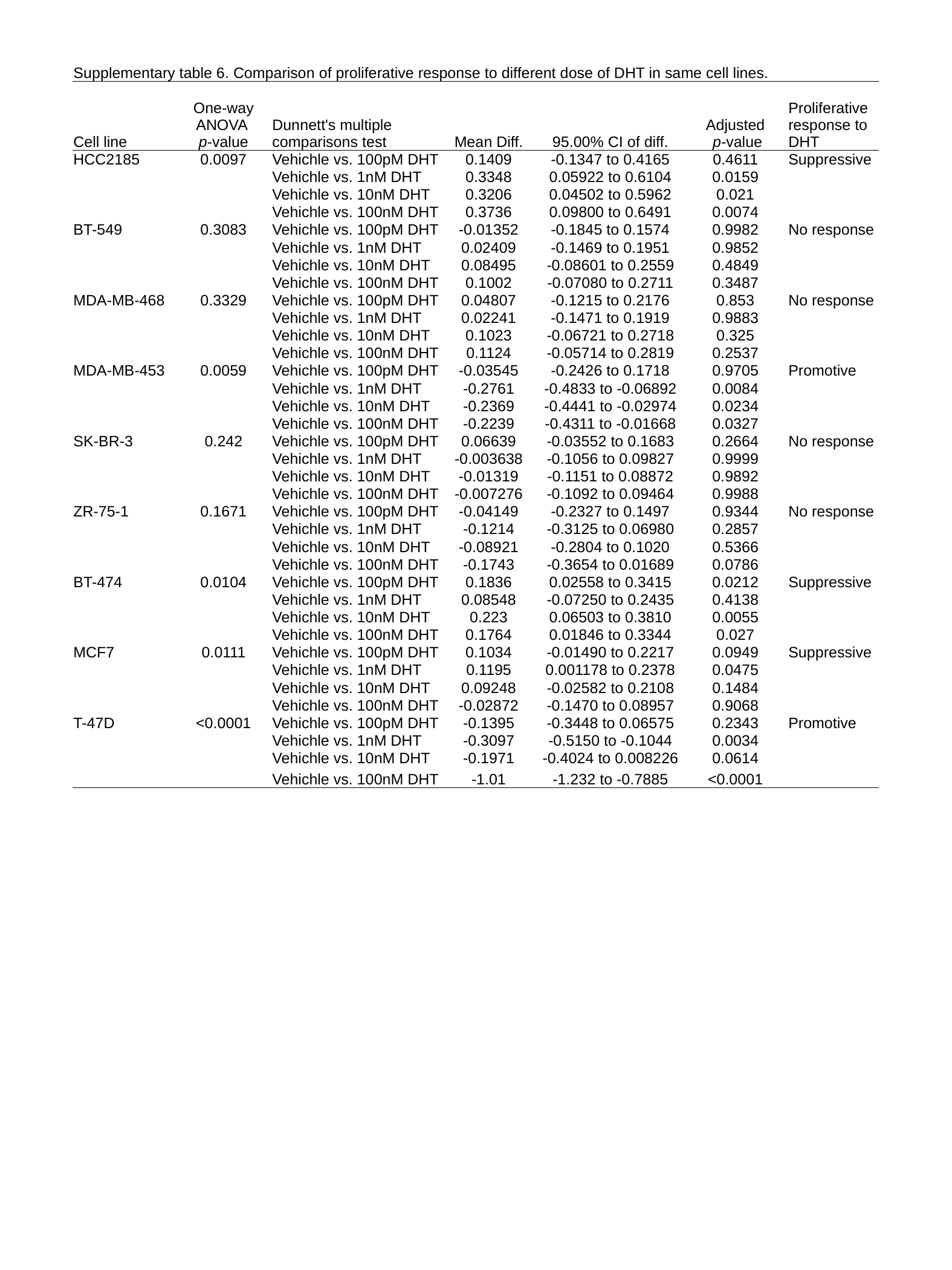

| Supplementary table 6. Comparison of proliferative response to different dose of DHT in same cell lines. | | | | | | |
| --- | --- | --- | --- | --- | --- | --- |
| | | | | | | |
| Cell line | One-way ANOVA p-value | Dunnett's multiple comparisons test | Mean Diff. | 95.00% CI of diff. | Adjusted p-value | Proliferative response to DHT |
| HCC2185 | 0.0097 | Vehichle vs. 100pM DHT | 0.1409 | -0.1347 to 0.4165 | 0.4611 | Suppressive |
| | | Vehichle vs. 1nM DHT | 0.3348 | 0.05922 to 0.6104 | 0.0159 | |
| | | Vehichle vs. 10nM DHT | 0.3206 | 0.04502 to 0.5962 | 0.021 | |
| | | Vehichle vs. 100nM DHT | 0.3736 | 0.09800 to 0.6491 | 0.0074 | |
| BT-549 | 0.3083 | Vehichle vs. 100pM DHT | -0.01352 | -0.1845 to 0.1574 | 0.9982 | No response |
| | | Vehichle vs. 1nM DHT | 0.02409 | -0.1469 to 0.1951 | 0.9852 | |
| | | Vehichle vs. 10nM DHT | 0.08495 | -0.08601 to 0.2559 | 0.4849 | |
| | | Vehichle vs. 100nM DHT | 0.1002 | -0.07080 to 0.2711 | 0.3487 | |
| MDA-MB-468 | 0.3329 | Vehichle vs. 100pM DHT | 0.04807 | -0.1215 to 0.2176 | 0.853 | No response |
| | | Vehichle vs. 1nM DHT | 0.02241 | -0.1471 to 0.1919 | 0.9883 | |
| | | Vehichle vs. 10nM DHT | 0.1023 | -0.06721 to 0.2718 | 0.325 | |
| | | Vehichle vs. 100nM DHT | 0.1124 | -0.05714 to 0.2819 | 0.2537 | |
| MDA-MB-453 | 0.0059 | Vehichle vs. 100pM DHT | -0.03545 | -0.2426 to 0.1718 | 0.9705 | Promotive |
| | | Vehichle vs. 1nM DHT | -0.2761 | -0.4833 to -0.06892 | 0.0084 | |
| | | Vehichle vs. 10nM DHT | -0.2369 | -0.4441 to -0.02974 | 0.0234 | |
| | | Vehichle vs. 100nM DHT | -0.2239 | -0.4311 to -0.01668 | 0.0327 | |
| SK-BR-3 | 0.242 | Vehichle vs. 100pM DHT | 0.06639 | -0.03552 to 0.1683 | 0.2664 | No response |
| | | Vehichle vs. 1nM DHT | -0.003638 | -0.1056 to 0.09827 | 0.9999 | |
| | | Vehichle vs. 10nM DHT | -0.01319 | -0.1151 to 0.08872 | 0.9892 | |
| | | Vehichle vs. 100nM DHT | -0.007276 | -0.1092 to 0.09464 | 0.9988 | |
| ZR-75-1 | 0.1671 | Vehichle vs. 100pM DHT | -0.04149 | -0.2327 to 0.1497 | 0.9344 | No response |
| | | Vehichle vs. 1nM DHT | -0.1214 | -0.3125 to 0.06980 | 0.2857 | |
| | | Vehichle vs. 10nM DHT | -0.08921 | -0.2804 to 0.1020 | 0.5366 | |
| | | Vehichle vs. 100nM DHT | -0.1743 | -0.3654 to 0.01689 | 0.0786 | |
| BT-474 | 0.0104 | Vehichle vs. 100pM DHT | 0.1836 | 0.02558 to 0.3415 | 0.0212 | Suppressive |
| | | Vehichle vs. 1nM DHT | 0.08548 | -0.07250 to 0.2435 | 0.4138 | |
| | | Vehichle vs. 10nM DHT | 0.223 | 0.06503 to 0.3810 | 0.0055 | |
| | | Vehichle vs. 100nM DHT | 0.1764 | 0.01846 to 0.3344 | 0.027 | |
| MCF7 | 0.0111 | Vehichle vs. 100pM DHT | 0.1034 | -0.01490 to 0.2217 | 0.0949 | Suppressive |
| | | Vehichle vs. 1nM DHT | 0.1195 | 0.001178 to 0.2378 | 0.0475 | |
| | | Vehichle vs. 10nM DHT | 0.09248 | -0.02582 to 0.2108 | 0.1484 | |
| | | Vehichle vs. 100nM DHT | -0.02872 | -0.1470 to 0.08957 | 0.9068 | |
| T-47D | <0.0001 | Vehichle vs. 100pM DHT | -0.1395 | -0.3448 to 0.06575 | 0.2343 | Promotive |
| | | Vehichle vs. 1nM DHT | -0.3097 | -0.5150 to -0.1044 | 0.0034 | |
| | | Vehichle vs. 10nM DHT | -0.1971 | -0.4024 to 0.008226 | 0.0614 | |
| | | Vehichle vs. 100nM DHT | -1.01 | -1.232 to -0.7885 | <0.0001 | |
